# Dietary docosahexaenoic acid supplementation inhibits acute pulmonary transcriptional and autoantibody responses to a single crystalline silica exposure in lupus-prone mice

**DOI:** 10.3389/fimmu.2024.1275265

**Published:** 2024-02-01

**Authors:** Preeti S. Chauhan, Abby D. Benninghoff, Olivia K. Favor, James G. Wagner, Ryan P. Lewandowski, Lichchavi D. Rajasinghe, Quan-Zhen Li, Jack R. Harkema, James J. Pestka

**Affiliations:** ^1^ Department of Food Science and Human Nutrition, Michigan State University, East Lansing, MI, United States; ^2^ Institute for Integrative Toxicology, Michigan State University, East Lansing, MI, United States; ^3^ Department of Animal, Dairy and Veterinary Sciences, Utah State University, Logan, UT, United States; ^4^ Department of Microbiology, Genetics, and Immunology, Michigan State University, East Lansing, MI, United States; ^5^ Department of Pharmacology and Toxicology, Michigan State University, East Lansing, MI, United States; ^6^ Department of Pathobiology and Diagnostic Investigation, Michigan State University, East Lansing, MI, United States; ^7^ Genecopoeia Inc, Rockville, MD, United States

**Keywords:** systemic lupus erythematosus, crystalline silica, omega-3 fatty acid, docosahexaenoic acid (DHA), inflammation, autoantibody, autoimmune disease, lung pathology

## Abstract

**Introduction:**

Workplace exposure to respirable crystalline silica (cSiO_2_) has been epidemiologically linked to lupus. Consistent with this, repeated subchronic intranasal cSiO_2_ instillation in lupus-prone NZBWF1 mice induces inflammation-/autoimmune-related gene expression, ectopic lymphoid tissue (ELT), autoantibody (AAb) production in the lung within 5 to 13 wk followed systemic AAb increases and accelerated onset and progression of glomerulonephritis within 13 to 17 wk. Interestingly, dietary docosahexaenoic acid (DHA) supplementation suppresses these pathologic effects, but the underlying molecular mechanisms remain unclear.

**Methods:**

This study aimed to test the hypothesis that dietary DHA supplementation impacts acute transcriptional and autoantibody responses in the lungs of female NZBWF1 mice 1 and 4 wk after a single high-dose cSiO_2_ challenge. Groups of mice were initially fed a control (Con) diet or a DHA-containing diet (10 g/kg). Cohorts of Con- and DHA-fed were subjected to a single intranasal instillation of 2.5 mg cSiO_2_ in a saline vehicle (Veh), while a Con-fed cohort was instilled with Veh only. At 1 and 4 wk post-instillation (PI), we compared cSiO_2_’s effects on innate-/autoimmune-related gene expression and autoantibody (AAb) in lavage fluid/lungs of Con- and DHA-fed mice and related these findings to inflammatory cell profiles, histopathology, cell death, and cytokine/chemokine production.

**Results:**

DHA partially alleviated cSiO_2_-induced alterations in total immune cell and lymphocyte counts in lung lavage fluid. cSiO_2_-triggered dead cell accumulation and levels of inflammation-associated cytokines and IFN-stimulated chemokines were more pronounced in Con-fed mice than DHA-fed mice. Targeted multiplex transcriptome analysis revealed substantial upregulation of genes associated with autoimmune pathways in Con-fed mice in response to cSiO_2_ that were suppressed in DHA-fed mice. Pathway analysis indicated that DHA inhibited cSiO_2_ induction of proinflammatory and IFN-regulated gene networks, affecting key upstream regulators (e.g., TNFα, IL-1β, IFNAR, and IFNγ). Finally, cSiO_2_-triggered AAb responses were suppressed in DHA-fed mice.

**Discussion:**

Taken together, DHA mitigated cSiO_2_-induced upregulation of pathways associated with proinflammatory and IFN-regulated gene responses within 1 wk and reduced AAb responses by 4 wk. These findings suggest that the acute short-term model employed here holds substantial promise for efficient elucidation of the molecular mechanisms through which omega-3 PUFAs exert protective effects against cSiO_2_-induced autoimmunity.

## 1 Introduction

The environment is a critical determinant of the onset and progression of autoimmune diseases in genetically predisposed individuals (1). Occupational exposure to respirable crystalline silica (cSiO_2_) is widespread in the construction, mining, and manufacturing industries (2) and has been etiologically linked to the development of lupus and other systemic autoimmune diseases (3, 4).

Relevant to human epidemiological studies, our laboratory has shown that four weekly intranasal instillations of 1 mg cSiO_2_ elicit, within 13 to 17 wk after the initial exposure, development of ectopic lymphoid tissue (ELT) in the lung, pulmonary autoantibody (AAb) production, systemic AAb elevation, and accelerated onset/progression of glomerulonephritis (5, 6). Using this subchronic model of environmental-triggered lupus, it was further determined that cSiO_2_ elicits dramatic changes in abundance of mRNA transcripts linked to innate and adaptive immune responses beginning at 5 wk after the initial cSiO_2_ dose that intensified as the autoimmune disease progressed (7). Affected genes were associated with the production and release of cytokines and chemokines, interferon (IFN) activity, complement activation, and adhesion molecules. Pulmonary transcriptomic signatures indicated elevations in numbers of neutrophils, macrophages, dendritic cells, B cells, and T cells that were consistent with the progression of autoimmunity.

Collectively, the above findings in the subchronic model point to the centrality of the pulmonary compartment for initiating autoimmunity and, ultimately, glomerulonephritis. Using this preclinical model to evaluate potential interventions, it was determined that dietary supplementation with the ω-3 polyunsaturated fatty acid (PUFA) docosahexaenoic acid (DHA) effectively prevents subchronic cSiO_2_ triggering of these and other lupus hallmarks (8–12). Thus, modulation of the cellular lipidome might be a valuable preventive for individuals who are genetically susceptible to lupus or other autoimmune diseases and/or at risk from exposures to environmental autoimmune triggers such as cSiO_2._

Even though cSiO_2_-triggered human autoimmunity can be modeled in lupus-prone mice by repeated exposure to the particle, the logistics of this subchronic preclinical model make it hard to dissect initial effects of cSiO_2_ in the lung mechanistically. Chauhan et al. (13) recently addressed these limitations by querying how a single intranasal instillation with 2.5 mg cSiO_2_, a dose routinely employed in mouse silicosis studies, influences acute inflammation and autoimmunity in the lungs of female NZBWF1 mice from 1 to 28 d post-instillation (PI). Acute cSiO_2_ exposure triggered inflammation and autoimmunity in disease in the lung as defined by prolonged inflammation and cell death, the appearance of proinflammatory cytokines and chemokines, lymphocyte recruitment, IFN response signature, B/T cell activation, and ELT development (13).

The findings that the short-term acute model efficiently recapitulates cSiO_2_’s subchronic effects suggest that it is amenable to mechanistic exploration into how dietary ω-3 PUFAs influence cSiO_2_-induced lupus. Towards this goal, we tested here the hypothesis that dietary DHA supplementation impacts acute transcriptional and autoantibody responses in the lungs of female NZBWF1 mice 1 and 4 wk after a single high dose cSiO_2_ challenge.

## 2 Materials and methods

### 2.1 Experimental design

**Figure 1:**
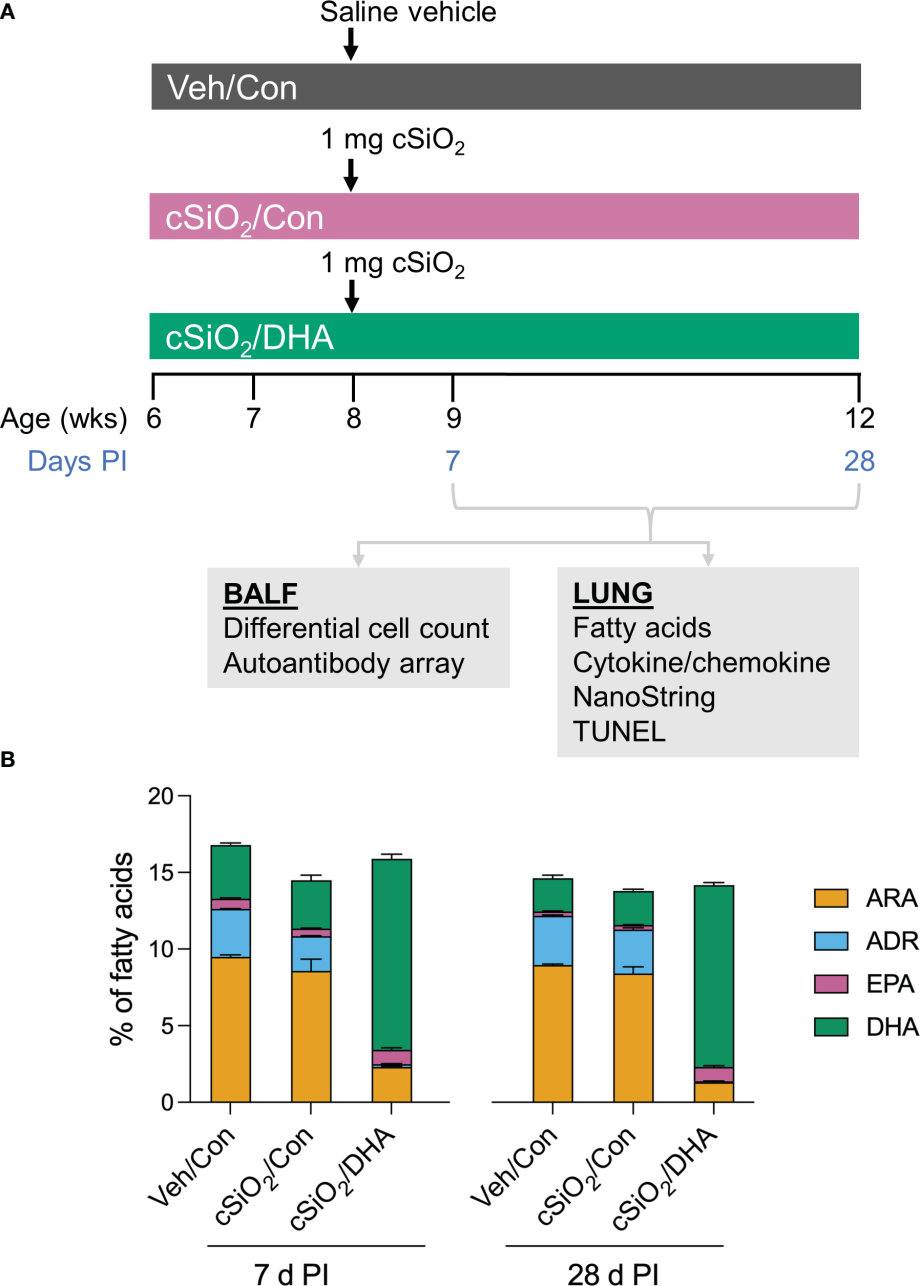
Experimental design **(A)** and confirmation of docosahexaenoic acid (DHA) incorporation into the lung phospholipids of DHA-fed mice **(B)**. **(A)** Female NZBWF1 mice were fed Con or DHA-enriched diets beginning at 6 wk of age (n=16 per group). At 8 wk of age, mice in the Veh/Con group were instilled with 25 μl PBS (Veh), whereas mice in groups cSiO_2_/Con and cSiO_2_/DHA were instilled with 25 μl PBS containing 2.5 mg cSiO_2_. Cohorts of mice were terminated at 7 d and 28 d post-instillation (PI) with cSiO_2_, and BALF and lung tissues were collected. BALF was used for differential cell count and AAb analysis. Lung tissue was used for fatty acid analysis, cytokine/chemokine ELISA, mRNA analyses, and TUNEL immunohistochemistry. **(B)** Consumption of a DHA-amended diet increases DHA and eicosapentaenoic acid (EPA) in lung phospholipids at the expense of adrenic acid (ADR) and arachidonic acid (ARA) at 7 d and 28 d post-cSiO_2_ instillation. Data reflect the percentage of total fatty acids in the lungs and are mean ± SEM (n=2).

**Figure 1A** shows the experimental design employed for this study. Experimental animal procedures were approved by the Michigan State University (MSU) Institutional Animal Care and Committee (AUF # PROTO201800113) at in accordance with the guidelines of the National Institute of Health (NIH). Six-week-old female NZBWF1 mice were obtained from Jackson Laboratories (Bar Harbor, ME) and housed under controlled conditions (humidity: 40-55%; lighting: 12-hour day/dark cycles; and temperature: 24 ± 2°C) in a MSU vivarium. Animals were randomly assigned to experimental groups, housed four per cage, and given free access to feed and water.

Groups of mice (n=16) were fed one of two isocaloric diets: 1) control (Con) American Institute of Nutrition (AIN) 93G diet (Dyets Inc. Bethlehem, PA) or 2) DHA-supplemented AIN-93G diet (**Supplementary Table 1**). Briefly, both diets contained 10 g/kg corn oil to ensure adequate basal essential fatty acids. The Con diet included 60 g/kg of high oleic safflower oil. The DHA diet contained 35 g/kg high oleic safflower oil and 25 g/kg DHASCO microalgal oil containing 40% (w/w) DHA (DHASCO, DSM Nutritional Products, Columbia, MD), yielding a final DHA concentration of 10 g/kg diet. This DHA dose is calorically equivalent to human consumption of 5 g/d of DHA, which is considered to be a safe intake (14). Diets were aliquoted in sealed bags and held at -20°C until use. Mice were fed a fresh diet every day to minimize fatty acid oxidation.

At age 8 wk, cohorts of Con- and DHA-fed mice were anesthetized with 4% (v/v) isoflurane and intranasally instilled with 2.5 mg cSiO_2_ suspended in 25 μl PBS vehicle (Veh) as previously described (13). In addition, separate Con-fed cohorts were instilled with 25 μl Veh only. Mice were fed their respective diets for 1 or 4 wk post-instillation (PI), and cSiO_2_’s effects on inflammatory cell profiles, histopathology, cell death, cytokines/chemokines, innate-/autoimmune-related gene expression, and autoantibody (AAb) production in lavage fluid/lungs were compared between Con- and DHA-fed mice. cSiO_2_ (Min-U-Sil 5, average particle size: 1.5–2.0 μm, Pennsylvania Sand Glass Corporation, Pittsburgh, PA, US) was acid-washed, oven-dried, and then suspended in sterile phosphate-buffered saline (PBS; Millipore Sigma). Suspensions were prepared fresh in PBS prior to use and sonicated and vortexed for an average of 1 min prior to each intranasal instillation. The cSiO_2_ dose of 2.5 mg has been frequently used in studies of silicosis in experimental mice (15–20). Based on the current NIOSH limit for respirable cSiO_2_ of 0.05 mg/m^3^/d and a human ventilation rate is 6.0 L/min, we estimate that workers would be exposed to 1433 mg respirable cSiO_2_ over 40 years of work (8 h/d for 5 d/wk). Further, assuming a ventilation rate of 0.03 L/min in mice, we estimate that the equivalent lifetime exposure to cSiO_2_ would be 8.3 mg in the mouse. Accordingly, the single acute cSiO_2_ dose used in this investigation represents approximately 30 percent of a human lifetime exposure to cSiO_2_ at the recommended NIOSH exposure limit.

### 2.2 Necropsy and sample collection

Mice were euthanized with an intraperitoneal injection of sodium pentobarbital (50 mg/kg body weight) and exsanguination through the inferior vena cava. Bronchoalveolar lavage fluid (BALF) was collected from whole lungs as described previously (21). Briefly, the trachea was exposed and cannulated, the lungs and heart were removed en bloc, and 0.8 ml of sterile saline was instilled through the cannulated trachea. BALF fluid was drawn from the lungs, the procedure was repeated, and collected BALF was pooled for analysis. The right lung lobe was removed, divided in half, and stored at -80°C for fatty acid and cytokine/chemokine analyses. The caudal lung lobe was then removed, stored in RNAlater (Thermo Fisher Scientific, Wilmington, DE) for a minimum of 24 h, and then frozen at -20°C until RNA analysis. The left lung lobe was intratracheally fixed for 1-2 h in 10% (v/v) neutral-buffered formalin (Fisher Scientific, Pittsburgh, PA) under constant pressure (30 cm H_2_O). Following fixation, the left lung lobe was processed to 30% (v/v) ethanol for histological preparation and subsequent analysis.

### 2.3 Fatty acid analysis

The fatty acid content of lung phospholipids was determined at Omega Quant, LLC (Sioux Falls, SD) by gas chromatography (GC) with flame ionization detection as previously described (22). Briefly, lung tissues were transferred into screw-cap glass vials, and an internal standard, 1,2-ditricosanoyl-sn-glycero-3-phosphocholine (di-C23:0 PL) sourced from Avanti Polar Lipids, USA, was introduced. A modified Folch extraction method was applied, with a portion of the organic layer subsequently applied to a thin-layer chromatography (TLC) plate and separated using a solvent mixture consisting of 8:2:0.15 (hexane: ethyl ether: acetic acid). Following the TLC procedure, the phospholipid band was collected from the plate and placed in a screw-cap glass vial containing methanol with 14% boron trifluoride from Sigma-Aldrich, St. Louis, MO. After a brief vortexing, the vial was heated to 100°C for 10 min. Post-heating, HPLC grade water, and hexane from EMD Chemicals, USA, were added sequentially, and the tubes were sealed, vortexed, and centrifuged to facilitate phase separation. Finally, the hexane layer underwent GC analysis using a GC2010 Gas Chromatograph equipped with a specific capillary column. Fatty acids were measured by comparison with a fatty acid standard mixture (GLC 782, NuCheck Prep) and an internal standard (C23:0 FAME, NuCheck Prep). Di-C23:0 PL was employed to calculate recovery efficiency for all fatty acids. The following 24 fatty acids (by class) were identified: saturated (14:0, 16:0, 18:0, 20:0, 22:0 24:0); cis monounsaturated (16:1, 18:1, 20:1, 24:1); trans (16:1, 18:1, 18:2), cis ω-6 polyunsaturated (18:2, 18:3, 20:2, 20:3, 20:4, 22:4, 22:5); cis ω-3 polyunsaturated (18:3, 20:5, 22:5, 22:6). Fatty acid concentrations were expressed as a percentage of total identified fatty acids.

### 2.4 BALF cell quantitation

Total leukocyte cell counts in BALF were quantified using a hemocytometer. Cells in BALF were immobilized on glass slides by centrifuging 150 µl of BALF at 40 × *g* for 10 min using Shandon Cytospin 3. Cytological slides were air-dried and then stained using Diff-Quick (Fisher Scientific). Differential cell counts for macrophages/monocytes, neutrophils, and lymphocytes in BALF were performed using morphological criteria on a total of 200 cells. The remaining BALF was centrifuged at 465 × *g* for 15 min, and the supernatant fraction was collected and stored at -80°C for AAb microarray.

### 2.5 Cytokine analysis by multiplexed ELISA

Half of the right lung was homogenized in cold RIPA lysis buffer (ThermoFisher) containing protease inhibitors. After homogenization, the samples were centrifuged at 12,000 × *g* for 15 min at 4°C. The supernatant was transferred to a prechilled 1.7 ml Eppendorf tube on ice. The total protein concentration in lung homogenate was determined using a BCA protein assay kit (ThermoFisher, Waltham, MA). The samples were adjusted to 1000 μg/ml with RIPA buffer. Lung homogenates were frozen and shipped overnight on dry ice to Eve Technologies (Calgary, Alberta, Canada) for analysis. Cytokines in lung homogenates were determined using a Mouse Cytokine Array/Chemokine Array 31-Plex (MD31) per manufacturer’s instructions.

### 2.6 Lung histopathology

Formalin-fixed left lung lobes were sectioned into four transverse lung blocks (~2 mm each). Tissue blocks were embedded in paraffin, and tissues were cut at 5 µm thickness, deparaffinized, and stained with hematoxylin and eosin (H&E). Light microscopic examination was performed by a board-certified veterinary pathologist (JRH) without knowledge of individual animal exposure.

### 2.7 IHC detection and quantification of B and T cells in lung tissue

Paraffin-embedded lung sections were immunohistochemically stained for CD45R^+^ B-cells and CD3^+^ T-cells (6, 23). Slides containing IHC-stained tissues were scanned using the VS200 virtual slide scanner (Olympus, Hicksville, NY). Prior to morphometric analysis, scanned slides were randomly subsampled to obtain >100 images captured per slide at 20× magnification using NewCast software (Visiopharm, Hoersholm, Denmark). Morphometric analysis used to quantify CD45R^+^ B-cells and CD3^+^ T-cells were quantified using the STEPanizer 1.8 stereology tool (24). Lymphocyte densities were counted by overlaying a point grid on randomly subsampled images. The number of grid points overlayed with IHC-stained cells was divided by reference tissue area to ascertain the percentage of the total lung area having positive IHC staining.

### 2.8 TUNEL

TUNEL immunostaining was used to label dead cells in 5 µm thick formalin-fixed, paraffin-embedded lung tissues using the *In Situ* Cell Death Detection Kit, TMR red (Roche, 12156792910) to measure the double-stranded cleavage of DNA according to the manufacturer’s instructions. Sections were deparaffinized by incubating them in a 60°C incubator for 1 h, followed by 15 min of immersion in xylene with two changes. Rehydration was done with a graded series of ethanol (100%, 90%, 70%, and 50% (v/v) ethanol) twice for 10 min. Sections were washed with deionized water for 5 min two times, then stained with TUNEL according to the manufacturer’s instructions. After that, the sections were washed twice in PBS. Finally, the nuclei were stained with 4′,6-diamidino-2-phenylindole-DAPI by mounting them in ProlongTM gold antifade reagent with DAPI (Invitrogen, P36931) and stored in the dark. An Evos FL Auto 2 microscope (Invitrogen) was used to examine stained sections, and images of six random fields were examined at an objective 10× magnification for analysis. ImageJ (https://imagej.nih.gov/ij/) was used to measure TUNEL positivity lung tissue images. The mean fluorescence within a region of interest (ROI) was measured and normalized by the intensity of DAPI fluorescence.

### 2.9 NanoString multiplex autoimmune gene profiling

RNA was extracted from 7 d and 28 d PI from caudal lung tissue (n=4) using Tri Reagent (Sigma Aldrich, St. Louis, MO) and TissueLyser II (Qiagen). Extracted RNA was purified using a Zymo RNA Clean and Concentrator Kit with DNase according to the manufacturer’s instructions (Zymo Research, Irvine, CA, cat no- R1017). The resulting RNA was dissolved in nuclease-free water and quantified using a Qubit fluorometer (Thermo Fisher Scientific). RNA integrity was assessed using a Qubit fluorometer (Agilent Technologies) at the MSU Genomics Core. Samples with RNA integrity >8 were analyzed using NanoString Autoimmune Gene Expression array (Cat # XT-CSO-MAIPI1-12) at the MSU Genomics Core. Assays were carried out using NanoString Technologies nCounter MAX system, sample preparation station, and digital analyzer per manufacturer’s instructions. Data were processed using nSolver 4.0 with the Advanced Analysis Module v2.0 as described previously (8). The background was subtracted using the eight negative controls included with the module. Gene counts under a threshold of 2σ of the mean background signal were not included in the subsequent analysis. Normalized linear counts and the calculated log_2_ ratio data are provided in **Supplementary File 1**. Differential gene expression analyses were performed as previously described (8). Three pairwise comparisons within each time point (7 d and 28 d) were determined as follows: cSiO_2_/Con vs. Veh/Con, cSiO_2_/DHA vs. Veh/Con, and cSiO_2_/DHA vs. cSiO_2_/Con. Statistically significant differences in gene expression were defined as a 1.5-fold change in expression (log_2_ >0.58 or <-0.58) with the Benjamini-Hochberg false discovery rate corrected (*p <*0.05); results of statistical analyses for all pairwise comparisons are provided in **Supplementary File 2**. BioVenn was used to create Venn diagrams of significant differentially expressed genes (25) using log_2_ transcript count data for differentially expressed genes (DEGs). ClustVis (26) was used to perform unsupervised hierarchical cluster analyses (HCC) with Euclidean distance with the average clustering method and principal component analyses (PCA) (https://biit.cs.ut.ee/clustvis/) using the SVD method with imputation.

### 2.10 Network analysis

Protein-protein interaction (PPI) network analyses of cSiO_2_- DHA-regulated genes were performed using STRING database version 11.5 (http://string-db.org/). This software facilitates critical assessment and integration of PPIs from multiple resources, including direct (physical) and indirect (functional) associations. Commonly upregulated DEGs were uploaded to STRING for the PPI network with the highest confidence (interaction score > 0.7) and Markov clustering (MCL) set to 3.

### 2.11 Upstream regulator network analysis (IPA analysis)

QIAGEN’s Ingenuity® Pathway Analysis (IPA®, QIAGEN Redwood City, www.qiagen.com/ingenuity) software was used for upstream regulatory analysis (URA) for 7 d and 28 d PI. IPA is a bioinformatics software package that analyzes genomic, proteomic, and experimental studies based on expert-reviewed and updated curated literature searches. The log_2_ fold-change values from NanoString advanced analysis were submitted to IPA and subjected to upstream regulator analysis.

### 2.12 High throughput autoantibody microarray profiling

High-throughput profiling of IgG AAb for a wide range of autoantigens (AAgs) was performed at the Microarray and Immune Phenotyping Core Facility at The University of Texas Southwestern Medical Center utilizing AAg coated protein arrays as described previously (9, 27). BALF samples were initially treated with DNase I to remove free DNA. Then, samples were diluted 1:25 and incubated in protein array plates coated with 122 antigens and 6 controls. Antibody binding was detected with Cy3-conjugated anti-mouse IgG(1:2000, Jackson ImmunoResearch Laboratories, PA) and fluorescent images captured with a Genepix 4200A scanner (Molecular Devices, CA). GenePix 7.0 software was used to transfer fluorescent images to signal intensity values and background subtracted and normalized to internal controls for IgG. Processed signal intensity values for each AAb were reported as antibody score (Ab-score), which was expressed based on the normalized signal intensity and signal-to-noise ratio (SNR) using the formula:


$$\text{Ab}-\text{score}={\text{log}}_{2}\left(\text{NSI}\times \text{SNR}+1\right)$$

Normalized and unit variance-scaled Ab-score values are represented in heat maps.

### 2.13 Indirect ELISA for AAbs to killed macrophage supernatants and purified nucleosomes

cSiO_2_- and staurosporine-killed RAW 264.7 macrophage (Mph) supernatants were prepared for dead cell AAg ELISAs. Briefly, RAW 264.7 cells were plated at 3.2×10^5^ cells/ml in RPMI 1640 media (0.25% FBS [v/v], 1% P/S [v/v]) in 100 mm cell culture dishes, exposed to cSiO_2_ (50 µg/ml) to induce apoptotic, pyroptotic, and necrotic death, then incubated for 20 h in a 37°C incubator (5% CO_2_). Additionally, RAW 264.7 cells were suspended at 1×10^7^ cells/ml in RPMI 1640 media (1% P/S [v/v]) and incubated with the apoptotic inducer staurosporine (1 µM) for 24 h in a 37°C incubator (5% CO_2_). Following incubation, the cell supernatants were collected and centrifuged for 10 min at 500 × *g*. Single-use aliquots were frozen at -20°C. IgG AAbs targeting cSiO_2_-killed Mph supernatant, staurosporine-killed Mph supernatant, and purified nucleosome were measured using a previously described indirect ELISA method (28). Prior to conducting the assay, individual BALF samples and plasma samples from each experimental group (n = 8) were pooled together to produce one pooled BALF and plasma sample per group. Nunc-Immuno™ Maxisorp microplates (Thermo Fisher Scientific) were treated with poly-L-lysine (20 µg/ml) in PBS (pH 7.4) and incubated overnight at 4°C. The next day, microplates were washed with PBS three times and then blocked with blocking buffer (PBS, 2% [w/v] BSA, 0.05% [v/v] Tween 20) (300 µl/well) at room temperature for 2-3 h. Microplates were washed three times with PBS and then incubated with STS, SKC material, or 2.5 µg/ml nucleosome antigen (Arotec Diagnostics) diluted in PBS-BSA (0.1% [w/v] BSA and 0.05% [v/v] Tween 20) (50 µl/well) at room temperature for 1 h. Microplates were washed three times with PBS, 50 µl of BALF or plasma diluted 1:20 in PBS-BSA was added to corresponding wells, and microplates were incubated at room temperature for 1 h. During sample addition, a standard curve was established by preparing a 2-fold dilution series of mouse anti-dsDNA antibodies (EMD Millipore Corporation, Temecula, CA), beginning by diluting 0.625 µl of the antibody stock into 1 ml of PBS-BSA. The preliminary dilution of the stock antibody marked the upper limit of the standard curve, which was assigned a value of 2000 arbitrary units (U). After standard antibody and sample incubation, microplates were washed three times with PBS and then treated with 1:5000 goat anti-human IgG Fc HRP-conjugated detection antibody (Southern Biotech, Birmingham, AL) (50 µl/well) at room temperature for 1 h. Microplates were washed three times with PBS and then treated for 20 min in the dark with K-Blue^®^ Advanced Plus TMB Substrate (Neogen) (50 µl/well). Sample absorbances were subsequently quantified using a FilterMax F3 Multimode plate reader (Molecular Devices, San Jose, CA) set to a wavelength of 650 nm.

### 2.14 Data analysis

The goal was to determine how DHA altered responses in a disease model (i.e., genetically predisposed animals exposed to the environmental trigger, cSiO_2_). Thus, the Veh/Con mice provided a negative control, while the Veh/cSiO_2_ mice provided a positive control. All statistical analyses were conducted using GraphPad Prism version 8 for Windows (GraphPad Software, La Jolla California USA, www.graphpad.com). To identify outliers, Grubb’s outlier test (Q = 1%) was used. The Shapiro-Wilk test was employed to determine data normality. The Kruskal-Wallis nonparametric test was used on data that did not meet normality and/or variance assumptions required for a parametric test, followed by Dunn’s *post-hoc* test. Normal data with equal variance were assessed using standard one-way ANOVA followed by Tukey’s *post-hoc* test or standard two-way ANOVA followed by Sidak’s *post-hoc* test, as appropriate. Data are presented as the mean ± standard error of the mean (SEM). A *p*-value ≤ 0.05 was considered statistically significant.

## 3 Results

### 3.1 Dietary DHA supplementation skews the balance of lung lipidome towards ω-3 PUFAs

The incorporation of ω-3 PUFAs into the lung tissue of DHA-fed mice was confirmed using GC (**Table 1**). The most abundant fatty acids in lung tissue of Con- and DHA-fed mice were i) saturated fatty acids palmitic acid (PA, C16:0), stearic acid (SA, C18:0), ii) the monosaturated fatty acid oleic acid (OA, C18:1ω9), and iii) the PUFAs linoleic acid (LA, C18:2ω6), arachidonic acid (ARA, C20:4ω6), adrenic acid (ADR, C22:4ω6), and DHA (C22:6ω3). cSiO_2_ instillation did not influence PUFA profiles in Con-fed mice. Replacing high-oleic safflower oil in the AIN-93G diet with DHA-rich algal oil increased the incorporation of DHA at the expense of OA, ARA, and ADR (**Figure 1B**). When expressed as percent total fatty acids, the Σ ω-3 PUFA rose from 3% in Con-fed mice to 14% in the DHA-fed mice.

**Table 1:** Lung tissue fatty acid content as determined by GLC.

Common Name	Chemical Formula	Veh/Con 7 d PI	cSiO_2_/Con 7 d PI	cSiO_2_/DHA 7 d PI	Veh/Con 28 d PI	cSiO_2_/Con 28 d PI	cSiO_2_/DHA 28 d PI
Myristic	C14:0	0.88 ± 0.05	1.63 ± 0.05	2.12 ± 0.45	1.12 ± 0.04	1.24 ± 0.05	3.01 ± 0.17
Palmitic	C16:0	32.90 ± 1.98	38.47 ± 3.06	40.05 ± 5.30	30.11 ± 0.38	31.68 ± 0.77	50.13 ± 1.71
Palmitolaidic	C16:1ω7t	0.085 ± 0.015	0.085 ± 0.005	0.075 ± 0.005	0.08 ± 0.01	0.075 ± 0.005	0.08 ± 0.00
Palmitoleic	C16:1ω7	3.34 ± 0.42	5.10 ± 0.68	5.95 ± 0.39	4.83 ± 0.16	5.87 ± 0.34	6.13 ± 0.32
Stearic	C18:0	14.15 ± 1.53	9.34 ± 0.79	8.37 ± 1.36	10.25 ± 0.13	9.28 ± 0.30	5.84 ± 0.44
Elaidic	C18:1t	0.185 ± 0.005	0.11 ± 0.01	0.09 ± 0.01	0.145 ± 0.005	0.115 ± 0.005	0.08 ± 0.02
Oleic	C18:1ω9	21.99 ± 2.69	21.94 ± 4.02	18.06 ± 3.68	29.47 ± 0.52	29.00 ± 2.08	13.89 ± 0.89
Trans-Linoleic	C18:2ω6t	0.25 ± 0.00	0.18 ± 0.03	0.095 ± 0.005	0.25 ± 0.02	0.22 ± 0.01	0.075 ± 0.005
Linoleic	C18:2ω6	5.38 ± 0.69	5.73 ± 0.59	6.42 ± 0.48	5.67 ± 0.04	5.48 ± 0.27	3.59 ± 0.48
Arachidic	C20:0	0.20 ± 0.00	0.13 ± 0.02	0.10 ± 0.03	0.145 ± 0.005	0.13 ± 0.01	0.08 ± 0.01
γ-linolenic	C18:3ω6	0.14 ± 0.02	0.13 ± 0.01	0.075 ± 0.005	0.145 ± 0.005	0.12 ± 0.01	0.05 ± 0.00
Erucic	C20:1ω9	0.675 ± 0.005	0.46 ± 0.01	0.23 ± 0.07	0.63 ± 0.04	0.57 ± 0.04	0.15 ± 0.03
α-linolenic	C18:3ω3	0.06 ± 0.01	0.08 ± 0.04	0.14 ± 0.02	0.055 ± 0.005	0.08 ± 0.02	0.035 ± 0.005
Eicosadienoic	C20:2ω6	0.25 ± 0.01	0.185 ± 0.005	0.13 ± 0.01	0.185 ± 0.005	0.16 ± 0.01	0.07 ± 0.01
Behenic	C22:0	0.24 ± 0.02	0.13 ± 0.02	0.14 ± 0.03	0.17 ± 0.02	0.15 ± 0.02	0.115 ± 0.005
Dihomo-γ-linolenic	C20:3ω6	1.06 ± 0.05	0.74 ± 0.06	0.63 ± 0.07	0.79 ± 0.02	0.74 ± 0.10	0.42 ± 0.05
Arachidonic	C20:4ω6	9.49 ± 0.13	8.57 ± 0.76	2.30 ± 0.06	8.95 ± 0.08	8.41 ± 0.43	1.31 ± 0.06
Lignoceric	C24:0	0.22 ± 0.01	0.15 ± 0.02	0.23 ± 0.05	0.185 ± 0.005	0.17 ± 0.02	0.24 ± 0.01
Eicosapentaenoic	C20:5ω3	0.05 ± 0.02	0.08 ± 0.01	0.78 ± 0.01	0.035 ± 0.005	0.05 ± 0.02	1.44 ± 0.10
Nervonic	C24:1ω9	0.40 ± 0.01	0.33 ± 0.03	0.32 ± 0.02	0.38 ± 0.02	0.38 ± 0.03	0.36 ± 0.02
Adrenic	C22:4ω6	3.13 ± 0.005	2.26 ± 0.04	0.18 ± 0.03	3.22 ± 0.04	2.86 ± 0.12	0.06 ± 0.01
Docosapentaenoic ω-6	C22:5ω6	0.73 ± 0.06	0.49 ± 0.05	0.06 ± 0.00	0.72 ± 0.08	0.69 ± 0.05	0.03 ± 0.00
Docosapentaenoic ω-3	C22:5ω3	0.66 ± 0.05	0.50 ± 0.03	0.94 ± 0.13	0.29 ± 0.03	0.30 ± 0.01	0.93 ± 0.07
Docosahexaenoic	C22:6ω3	3.49 ± 0.14	3.15 ± 0.33	12.47 ± 0.30	2.16 ± 0.20	2.21 ± 0.13	11.87 ± 0.17
Σ SFA		48.59 ± 3.51	49.87 ± 3.97	51.02 ± 4.27	41.98 ± 0.58	42.65 ± 1.17	59.42 ± 1.42
Σ MUFA		26.69 ± 3.12	28.02 ± 4.67	24.72 ± 3.39	35.55 ± 0.61	36.01 ± 2.34	20.69 ± 0.64
Σ PUFA		24.73 ± 0.38	22.11 ± 0.70	24.24 ± 0.88	22.48 ± 0.03	21.34 ± 1.17	19.88 ± 0.78
Σ ω-6 PUFA		20.45 ± 0.58	18.30 ± 0.37	9.90 ± 0.53	19.94 ± 0.27	18.70 ± 0.99	5.60 ± 0.63
Σ ω-3 PUFA		4.42 ± 0.18	3.95 ± 0.35	14.42 ± 0.34	2.68 ± 0.24	2.76 ± 0.19	14.33 ± 0.15
ω3/ω6 ratio		0.22 ± 0.02	0.22 ± 0.01	1.46 ± 0.04	0.13 ± 0.01	0.15 ± 0.00	2.59 ± 0.26

Data are presented as percent of total fatty acids (mean ± SEM, n = 2/gp) as measured by GLC.

(% of total fatty acids, mean ± SEM).

### 3.2 DHA consumption influences acute cSiO_2_-induced changes in cellularity and cytokines/chemokines in BALF but not lung histopathology

**Figure 2:**
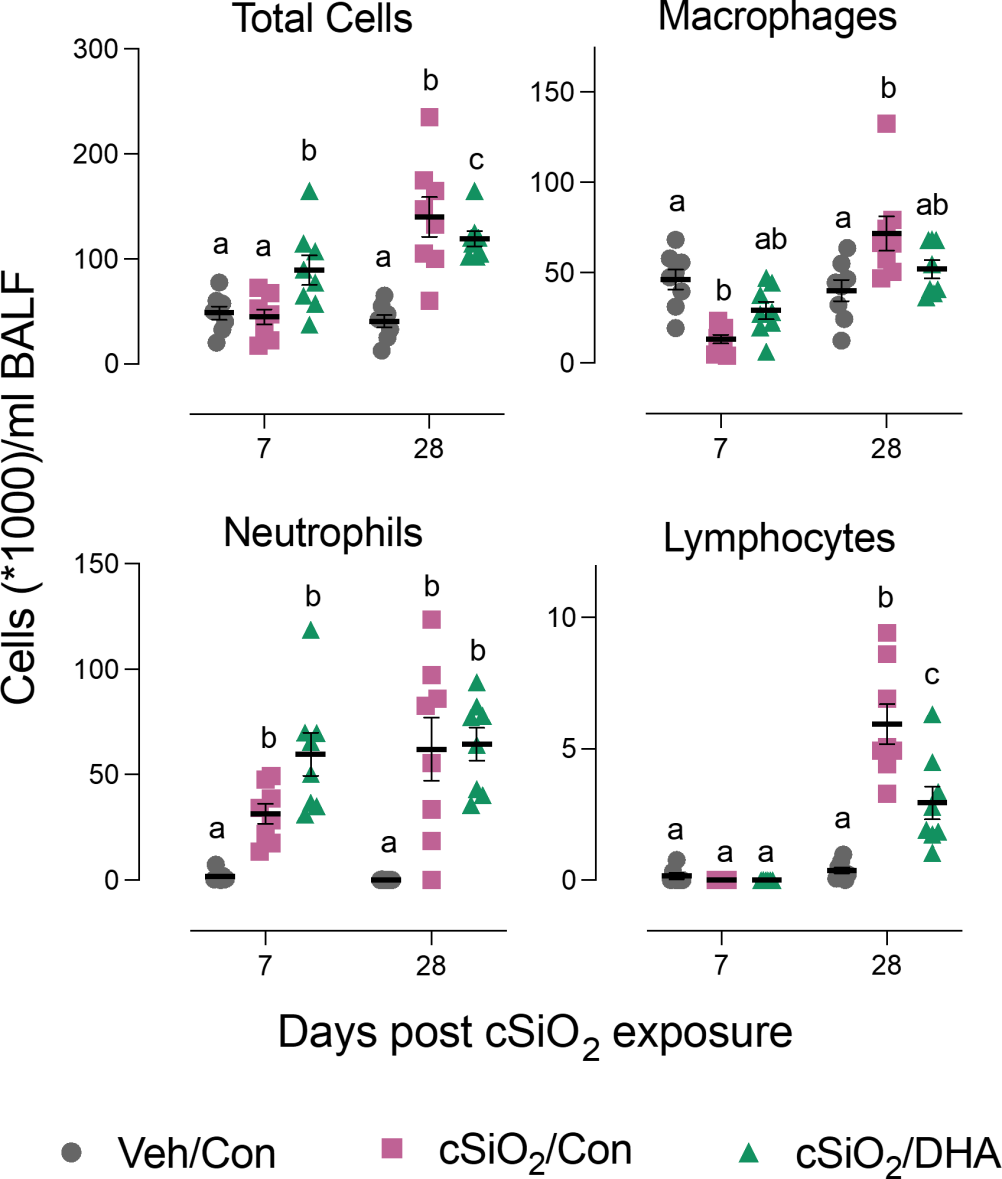
DHA supplementation suppresses cSiO_2_-induced total cell and lymphocyte accumulation in the bronchoalveolar lavage fluid. BALF was collected at 7 d and 28 d PI, and total cell, monocytes/macrophages, neutrophils, and lymphocyte counts were determined. Individual data are shown with the mean ± SEM (n=8). Within each time point, groups without the same letter are significantly different (p ≤ 0.05).

Acute cSiO_2_ exposure of Con-fed mice decreased the numbers of macrophages and increased neutrophil count at 7 d PI in the bronchoalveolar lavage fluid. DHA-fed mice treated with cSiO_2_ exhibited less macrophage loss (**Figure 2**). At 28 d PI, Con-fed mice treated with the particles exhibited elevations in total cells, macrophages, neutrophils, and lymphocytes indicative of extensive recruitment of these leukocyte populations. However, in DHA-fed mice, there was a reduction in total cell, and lymphocyte counts following cSiO_2_ treatment at 28 d PI. Thus, DHA supplementation subtly influenced cSiO_2_-induced inflammatory cell responses at both time points.

**Figure 3:**
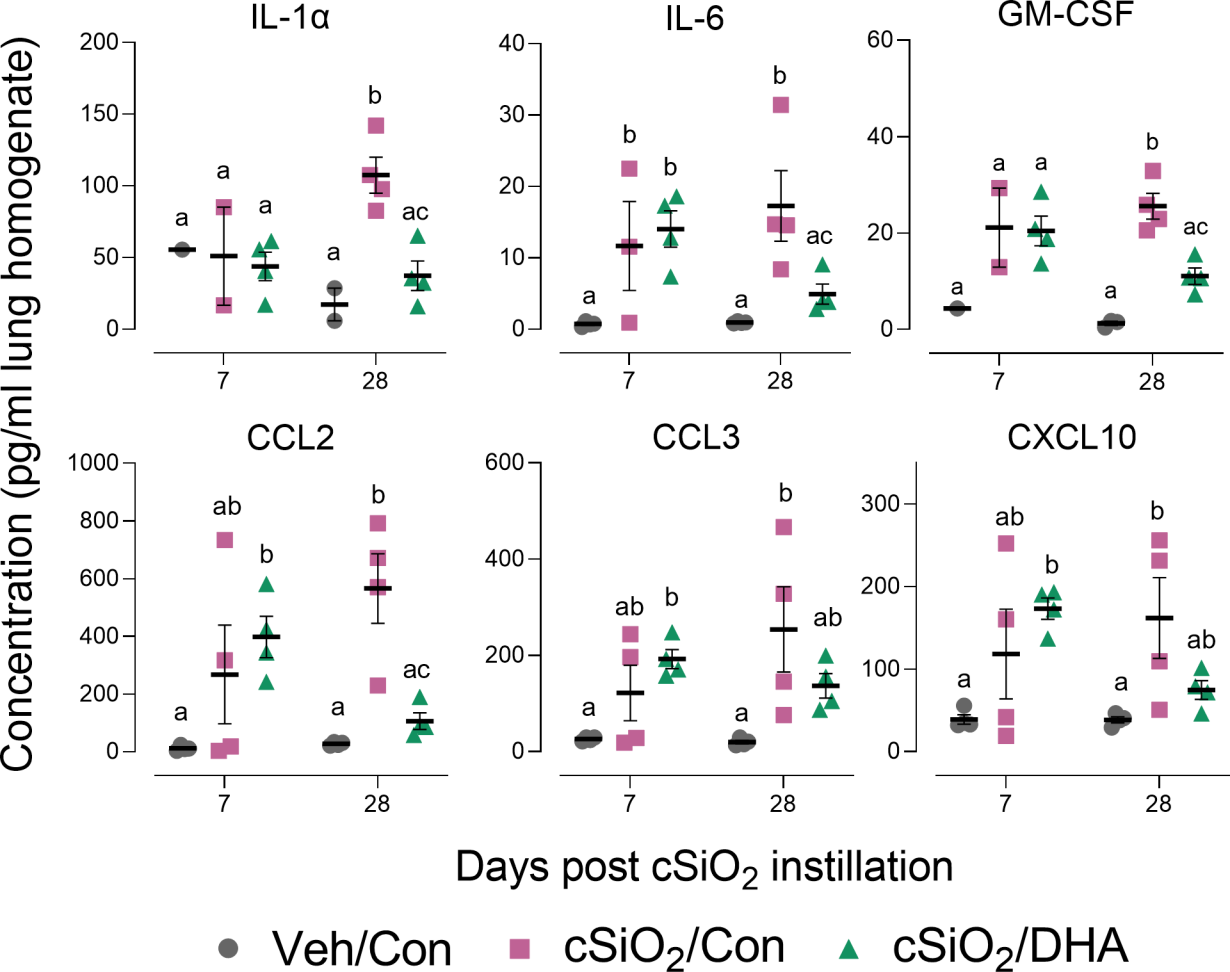
DHA supplementation inhibits cSiO_2_-induced cytokine and chemokine elevation in the lung tissue. Selected cytokines and chemokines were assessed in lung homogenates by multiplexed ELISA. Individual data are shown with the mean ± SEM (n=4). Within each time point, groups without the same letter are significantly different (p ≤ 0.05).

cSiO_2_ induced protein expression in the lung of representative inflammation-associated cytokines (IL-1α, IL-6, and GM-CSF) and IFN-stimulated chemokines (CCL2, CCL3, CXCL10) in the Con-fed cohort at 7 and/or 28 d PI (**Figure 3**). Consistent with BALF cellularity findings, these protein responses were markedly suppressed in DHA-fed mice at 28 d PI.

Histologic evaluation in lungs from Con-fed mice treated with cSiO_2_ indicated that there was mild to moderate acute neutrophilic inflammation and epithelial cell hyperplasia in the centriacinar region as well as proteinosis at 7 and 28 d PI (**Supplementary Figures 1A, B**). In addition, there was modest perivascular lymphocytic infiltration, especially around pulmonary veins, at 28 d PI. These histopathologic effects were not influenced by dietary DHA supplementation. The lymphocytic infiltrates in the lungs of cSiO_2_-treated Con-fed mice contained B and T cell aggregates (**Supplementary Figures 2A, B**). Relative amounts of cSiO_2_-induced B and T cell-positive cells did not significantly differ between Con- and DHA-fed mice. Accordingly, unlike the lavage counts and lung cytokine responses, cSiO_2_-induced lung histopathology and ELT development were unaffected by DHA.

### 3.3 DHA supplementation suppresses acute cSiO_2_-induced dead cell accumulation in the lung

**Figure 4:**
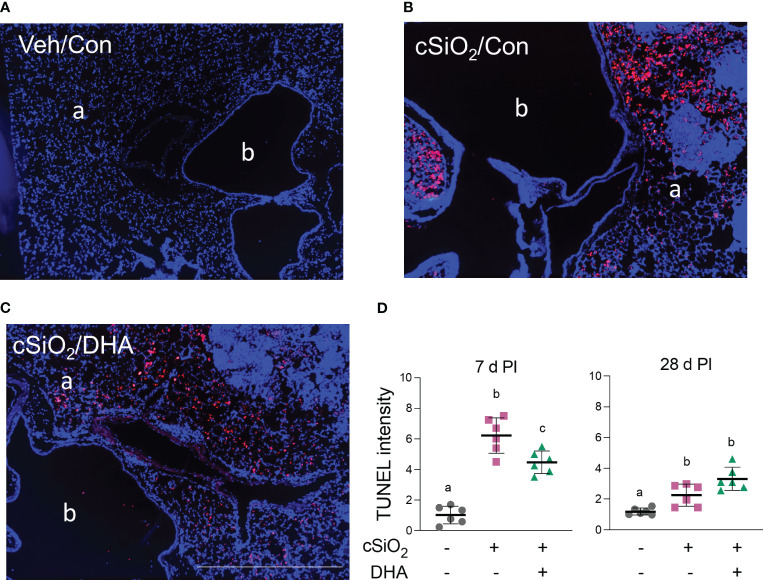
DHA intake attenuates cSiO_2_-induced cell death in the lung at 7 d PI. **(A–C)** TUNEL immunostaining of representative tissue sections from the left lung of **(A)** Veh/Con, **(B)** cSiO_2_/Con, and **(C)** cSiO_2_/DHA treated mice. a, alveolar parenchyma; b, bronchiolar airway. The scale bar indicates 200 µm. **(D)** Digital morphometry revealed less TUNEL positivity in the lungs of DHA-fed mice 7 d after cSiO_2_ treatment. Individual data are shown with the mean ± SEM (n=6). Within each time point, groups without the same letter are significantly different (p ≤ 0.05).

TUNEL staining of lung sections in Con-fed acutely exposed to cSiO_2_ revealed pronounced DNA fragmentation indicative of cell death at 7 d PI (**Figures 4A, B**). cSiO_2_-induced TUNEL^+^ tissue was significantly reduced in the DHA-fed mice, suggesting that the fatty acid suppressed dead cell accumulation (**Figures 4C, D**). While dead cells were still evident in cSiO_2_-treated mice at 28 d PI, the response was markedly less than 7 d PI and unaffected by DHA feeding.

### 3.4 DHA feeding suppresses acute cSiO_2_-triggering of multiple gene pathways associated with inflammation and autoimmunity

**Figure 5:**
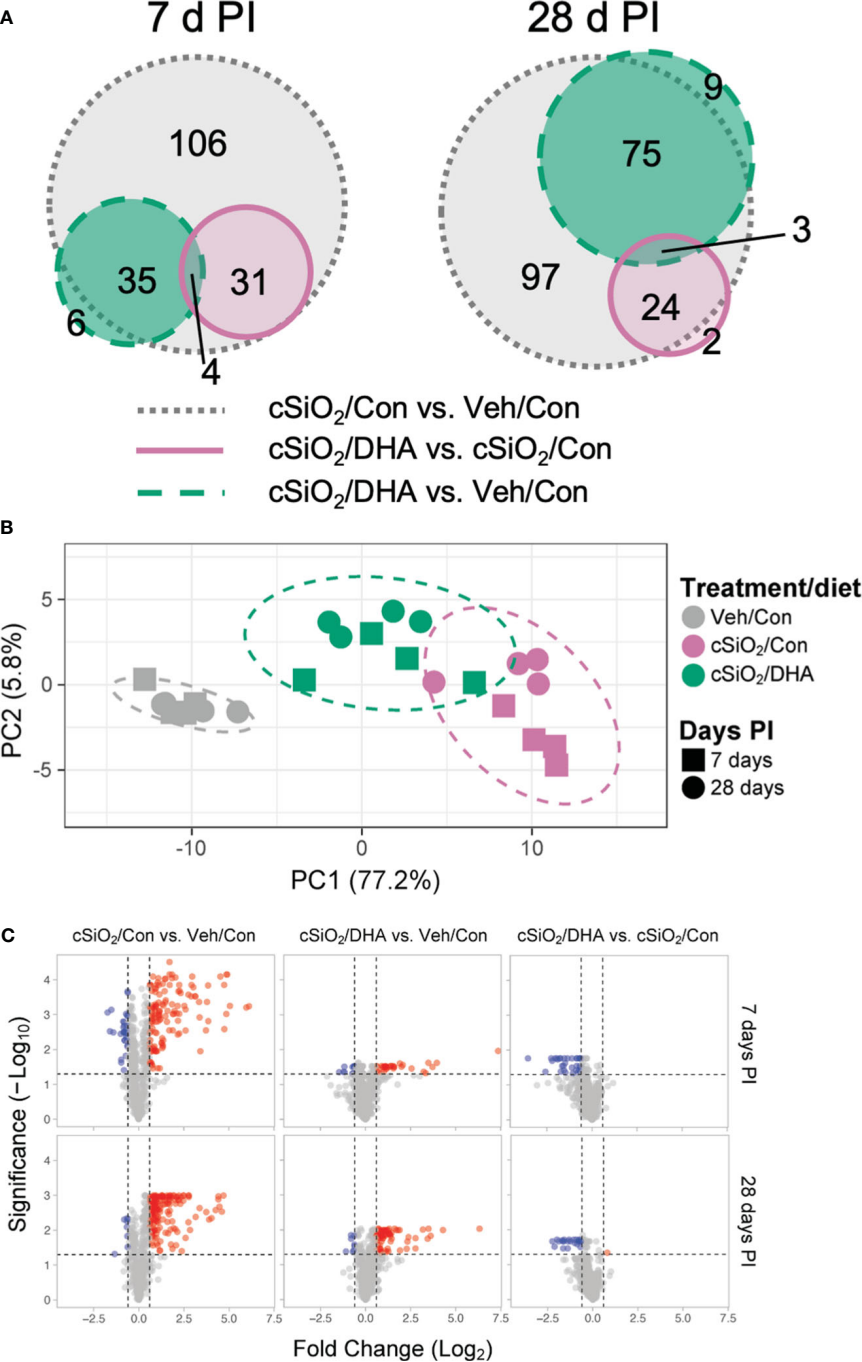
DHA supplementation impedes cSiO_2_-induced modulation of autoimmune-related genes in the lung. Lung RNA was subjected to NanoString analysis using the nCounter Autoimmune Gene Expression panel. **(A)** Proportional Venn diagrams depict numbers of genes differentially regulated in for all pairwise comparisons among treatment/diet groups (FDR *p <*0.05, 1.5-fold change) at 7 or 28 d PI. **(B)** Principal component analysis (PCA) of normalized transcript counts in lung tissue of Veh/Con, cSiO_2_/Con, and cSiO_2_/DHA treated mice at 7 or 28 d PI. Ellipses indicate the 95% confidence interval. **(C)** Volcano plots of differentially expressed genes (FDR *p <*0.05, 1.5-fold change) in lung tissues of mice for all pairwise comparisons among treatment/diet groups at 7 or 28 d PI. Red, genes up-regulated; blue, genes significantly down-regulated; gray, genes not significantly affected.

The effects of Con or DHA diets on cSiO_2_-induced gene responses in the lung at 7 and 28 d PI were compared using the NanoString Autoimmune Gene Expression array. We found that 115 and 130 genes were differentially regulated (FDR *q*<0.05, 1.5-fold change) in the lungs of cSiO_2_-instilled Con-fed mice at 7 d and 28 d PI, respectively (**Figure 5A**). In mice exposed to cSiO_2_ and fed DHA, 35 and 29 genes were differentially expressed at 7 or 28 d PI, respectively, as compared to the cSiO_2_-exposed mice fed Con diet. Principal component analysis indicated that the three experimental groups distinctly clustered by the treatment/diet group as opposed to the day PI (**Figure 5B**). Most DEGs affected by cSiO_2_ were upregulated, whereas genes affected by DHA treatment were nearly all suppressed compared to the cSiO_2_/Con group for either time point (**Figure 5C**).

**Figure 6:**
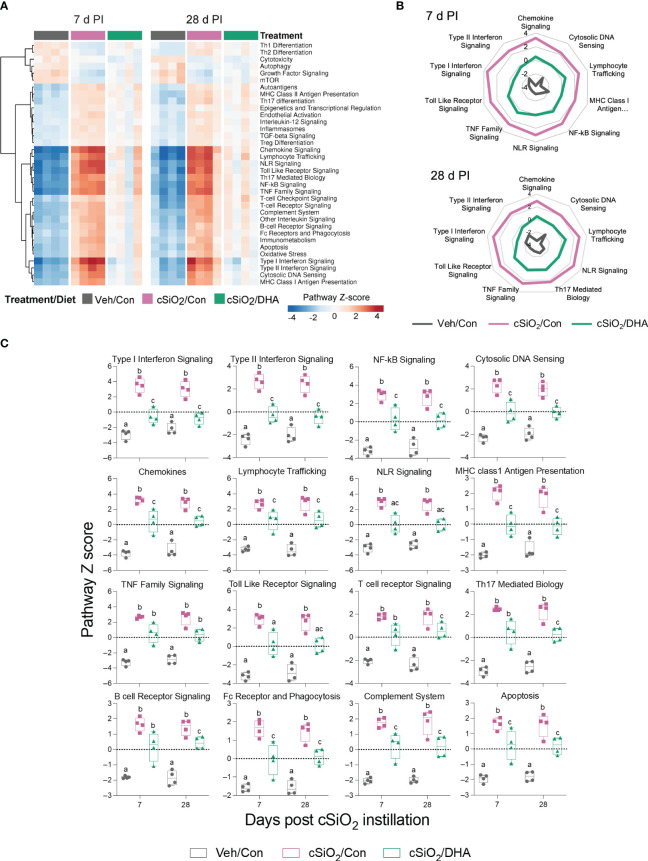
DHA consumption interferes with cSiO_2_-induced modulation of inflammatory- and autoimmune-related pathways in the lung. **(A)** Hierarchical clustering heatmap (Euclidean distance method) depicting pathway Z scores for inflammatory- and autoimmune-related pathways determined using nSolver analysis. **(B)** Radar and **(C)** Box plots of Z score data demonstrating DHA suppression of selected cSiO_2_-induced autoimmune pathways. Box plots represent the min to the max with the median (n=4). Within each time point, groups with different letters are significantly different (p ≤ 0.05).

When gene expression pathway scores were determined as the first principal component of the pathway genes’ normalized expression and standardized by Z scaling, acute cSiO_2_ instillation was found to induce numerous inflammation- and autoimmune-associated pathways at both 7 and 28 d PI that were significantly downregulated by DHA supplementation (**Figures 6A–C**). These pathways were associated with chemokine, TLR, NLR, TNF, B/T cell receptor signaling, Type 1 and 2 IFN-stimulation, cytosolic DNA sensing, lymphocyte trafficking, MHC class 1 antigen presentation, FcR and phagocytosis, complement, and apoptosis.

### 3.5 Acute cSiO_2_ exposure induces in Con-fed mice early and robust expression of Type 1 IFN-regulated and other genes that are ablated in DHA-fed mice

**Figure 7:**
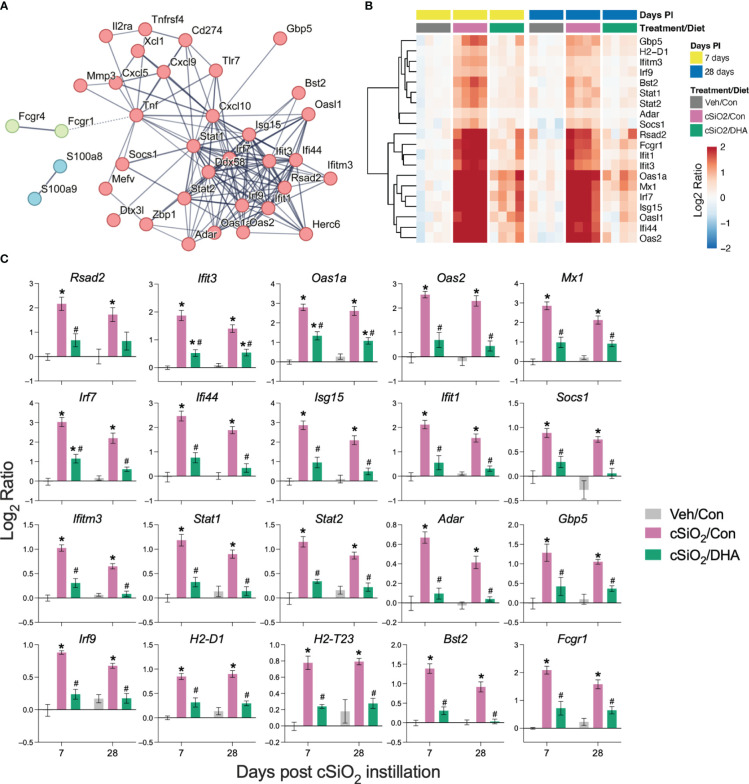
DHA supplementation suppresses cSiO_2_-induced IFN-regulated gene expression in the lung. Gene expression data were obtained using the autoimmune profile panel subjected to NanoString analysis. **(A)** Protein-protein interaction (PPI) network of commonly upregulated DEGs expressed was created using STRING. The interactions were visualized with a high confidence >0.7. Proteins were clustered using the MCL algorithm. IFN-regulated genes (red nodes) were among the top genes affected by DHA. **(B)** Hierarchical clustering heatmap (Euclidean distance method) showing cSiO_2_-induced IFN-regulated genes that are significantly affected (FDR *p*<0.05) by DHA. Values are shown as the log_2_ ratio with respect to 7 d PI, Veh/Con group. **(C)** DHA diet suppresses the expression of cSiO_2_-induced genes in the lungs. Data are shown as log_2_ ratio ± SEM (n=4) for cSiO_2_-exposed mice fed Con (cSiO_2_/Con) or DHA diet (cSiO_2_/DHA) for either time point with respect to the day 7 Veh/Con group. Within each time point, * indicates significantly different compared to time-matched Veh/Con or # indicates significantly different compared to time-matched cSiO_2_/Con (FDR *p ≤* 0.05).

PPI network mapping of upregulated DEGs revealed that Type 1 IFN-regulated genes were among the top cSiO_2_-induced genes affected by DHA (**Figure 7A**). The effects of cSiO_2_ and DHA on representative Type 1 IFN-regulated genes are shown as heat maps (**Figure 7B**) and bar charts (**Figure 7C**). Consistent with the network analysis, DHA significantly suppressed the cSiO_2_-induced upregulation of the Type 1 IFN-regulated genes, including those coding for interferon response factors (*Irf7* and *Irf9*), interferon-induced proteins (*Ifi35*, *Ifi44*, *Ifit1*, *Ifit3*, *Ifih1*, and *Ifitm3*), 2’-5’-oligoadenylate synthetases (*Oas1a* and *Oas2*), antiviral proteins (*Rsad2*, *Mx1*, and *Adar)*, and signal transducer and activator of transcription proteins (*Stat1* and *Stat2*).

**Figure 8:**
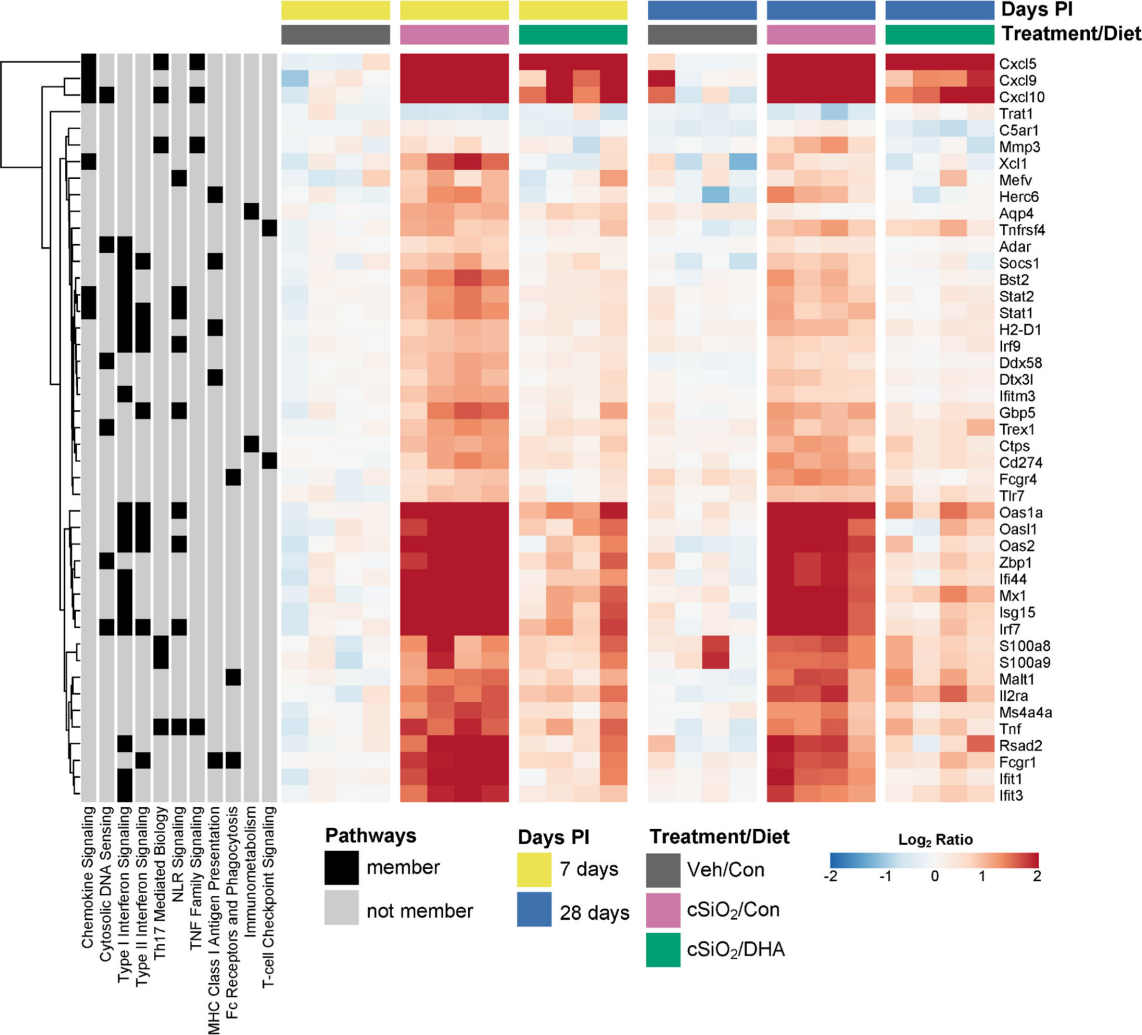
DHA supplementation suppresses cSiO_2_-triggered expression of diverse autoimmune-pathway-related genes. Hierarchical clustering heatmap (Euclidean distance method) shows the relative expression of genes that were differentially regulated by DHA at either 7 or 28 d PI. Values are shown as the log_2_ ratios of gene expression with respect to the 7 d PI, Veh/Con mice. Annotations to the left of the heatmap indicate membership of each gene in selected autoimmune pathways.

**Figure 8** illustrates other selected cSiO_2_-induced genes associated with TLR activation, DNA signaling, chemokines, Type 2 IFN response signatures, lymphocyte trafficking, MHC class 1 antigen presentation, and B and T cell activation that were attenuated by DHA supplementation.

### 3.6 DHA supplementation strongly influences top regulators of acute cSiO_2_-induced inflammatory and autoimmune gene expression

**Figure 9:**
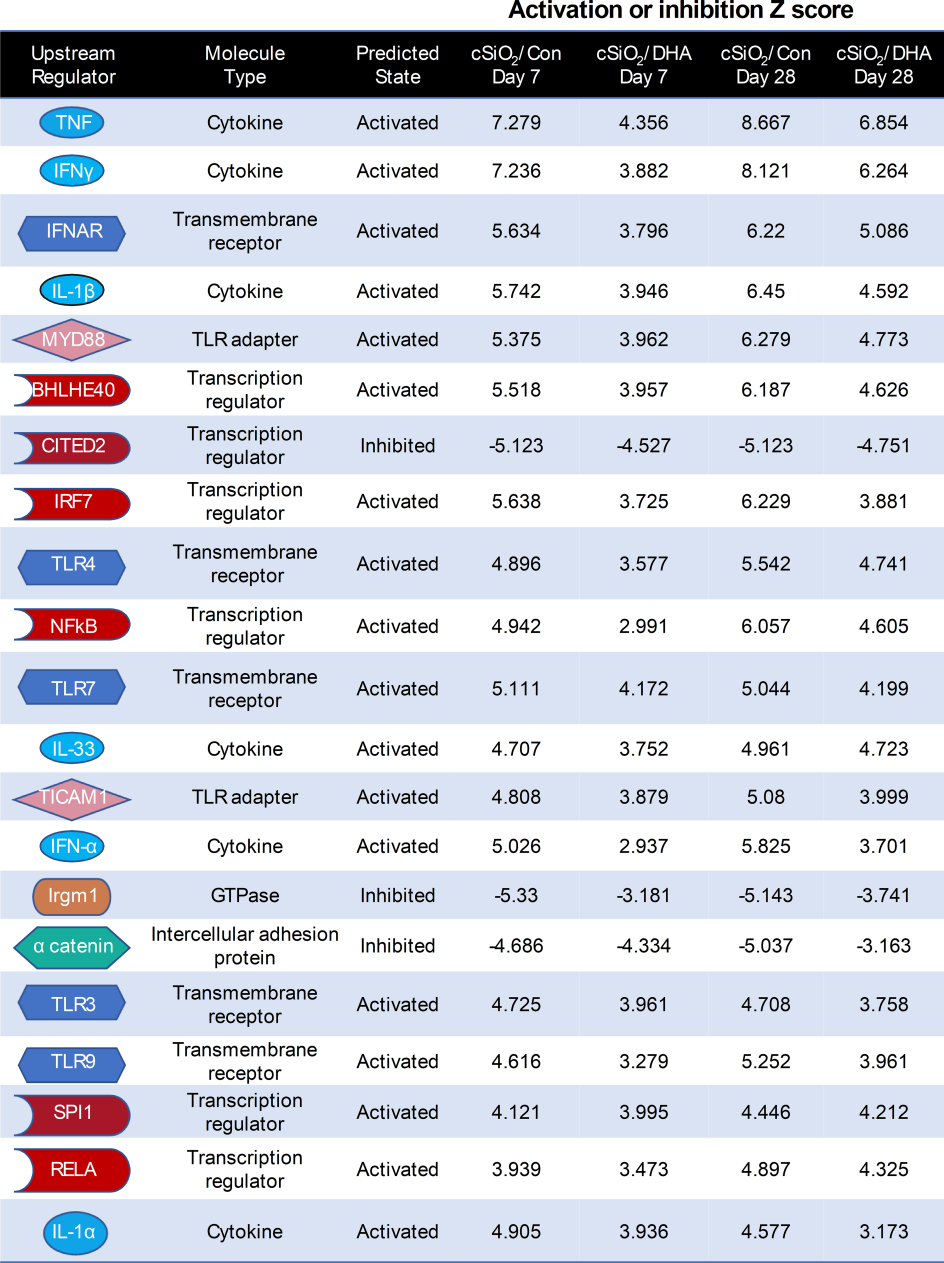
DHA intake quells responses to top cSiO_2_-induced upstream regulators at 7 and 28 d PI. Top upstream regulators in cSiO_2_/Con and cSiO_2_/DHA groups in the lung predicted by Ingenuity Pathway Analysis (IPA). A positive Z-score indicates activation and a negative Z-score indicates inhibition. Activation and inhibition scores in the cSiO_2_/Con group were markedly lower in the corresponding cSiO_2_/DHA for the same week.

Ingenuity Pathway Analysis (IPA) suggested that cSiO_2_ influences a multitude of upstream regulators of proinflammatory and IFN-regulated gene networks in Con-fed mice (**Figure 9**). Remarkably, DHA supplementation influenced the activation and inhibition Z scores for these regulators, many of which were discernable as early as 7 d after acute cSiO_2_ instillation. Top upstream regulators included cytokines (TNF, IL-1β, IL-1α, IFNγ, IFNα), transmembrane receptors (IFNAR, TLR-3, TLR4, TLR7, TLR9), TLR adapters (MYD88, TICAM1), transcription factors (BHLHE40, CITED2, IRF7, NFκB, SPL1, and RELA) and a GTPase (Irgm1). Representative gene networks affected following acute cSiO_2_ treatment at 7 d PI included TNFα, IL-1β, IFNAR, and IFNγ (**Supplementary Figures 3–7**).

### 3.7 Diverse AAb production in the lung induced by cSiO_2_ is suppressed by DHA intake

**Figure 10:**
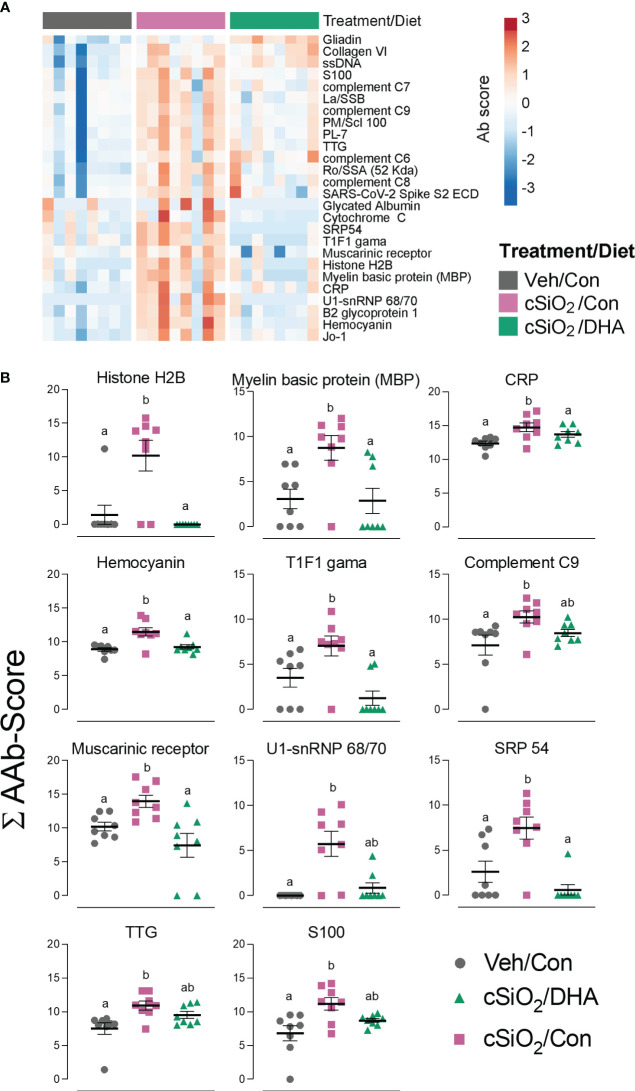
DHA intake suppresses cSiO_2_-induced autoantibody (AAb) responses in the lung. **(A)** Hierarchical clustering (Euclidean distance method) heat map of Ab-score values (row centered, variance stabilized) for expression of selected AAbs of the IgG isotype in BALF at 28 d PI as determined by microarray. **(B)** ∑Ab-scores of selected AAbs of the IgG isotype in BALF at 28 d PI. Data are mean ± SEM (n=8). Groups without the same letter are significantly different (p ≤ 0.05).

**Figure 11:**
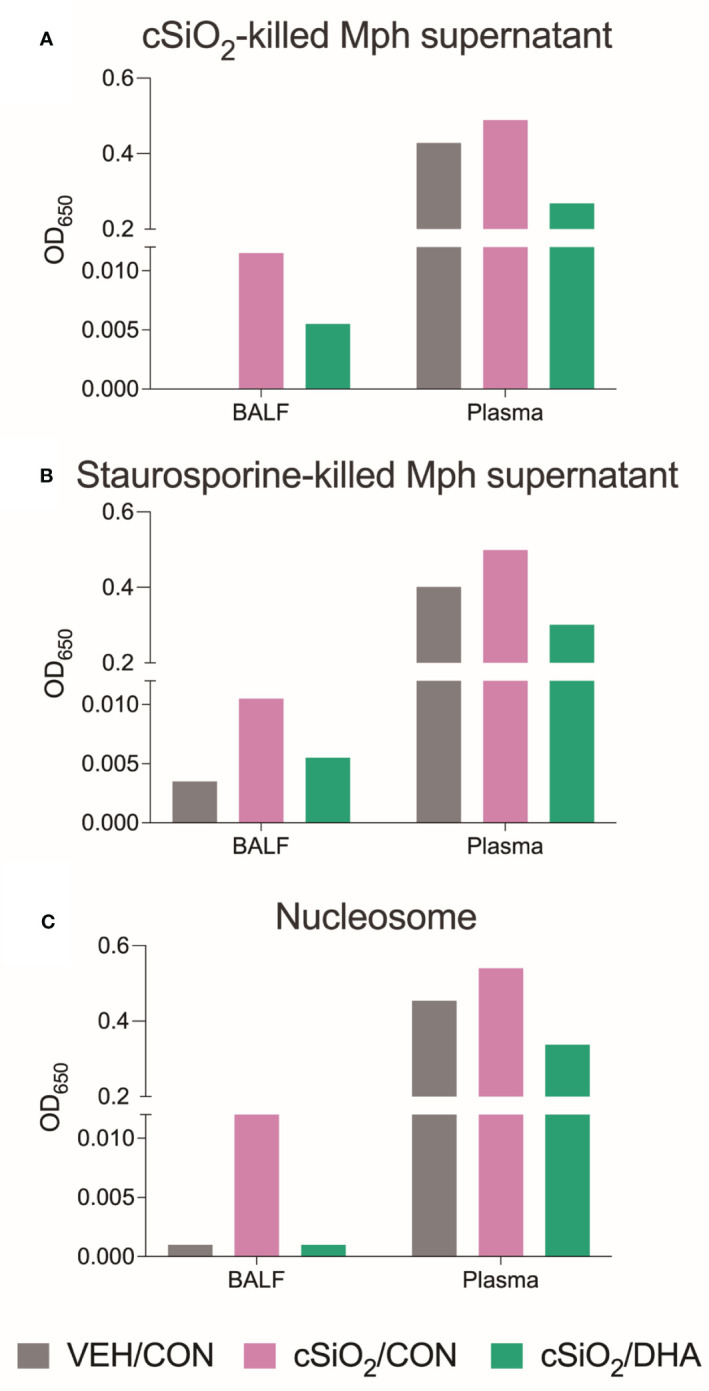
DHA supplementation suppressed IgG AAbs specific to cSiO_2_-killed cells **(A)**, staurosporine-killed cells **(B)**, and nucleosomes **(C)** in BALF and plasma. BALF and plasma of Veh/Con, cSiO_2_/Con, and cSiO_2_/DHA mice sacrificed at 28 d PI were pooled, and IgG AAbs were measured by ELISA.

Using the AAg protein microarray of BALF collected at 28 d PI, we found that cSiO_2_ induced a broad range of AAbs with specificities for nuclear, ribosomal, mitochondrial, and complement proteins in Con-fed mice (**Figures 10A, B**, **Supplementary Figure 8**). Consistent with the observed reduction in TUNEL positivity at 7 d PI in DHA-fed mice, we found that AAb responses were markedly suppressed in the DHA cohort. Finally, ELISA of both BALF and plasma indicated that DHA suppresses cSiO_2_-induced increases in IgG Abs reactive with cSiO_2_-killed macrophage (Mph) supernatant (**Figure 11A**), staurosporine-killed Mph supernatant (**Figure 11B**), and purified nucleosome (**Figure 11C**) in both BALF and plasma of lupus-prone NZBWF1 mice.

## 4 Discussion

**Figure 12:**
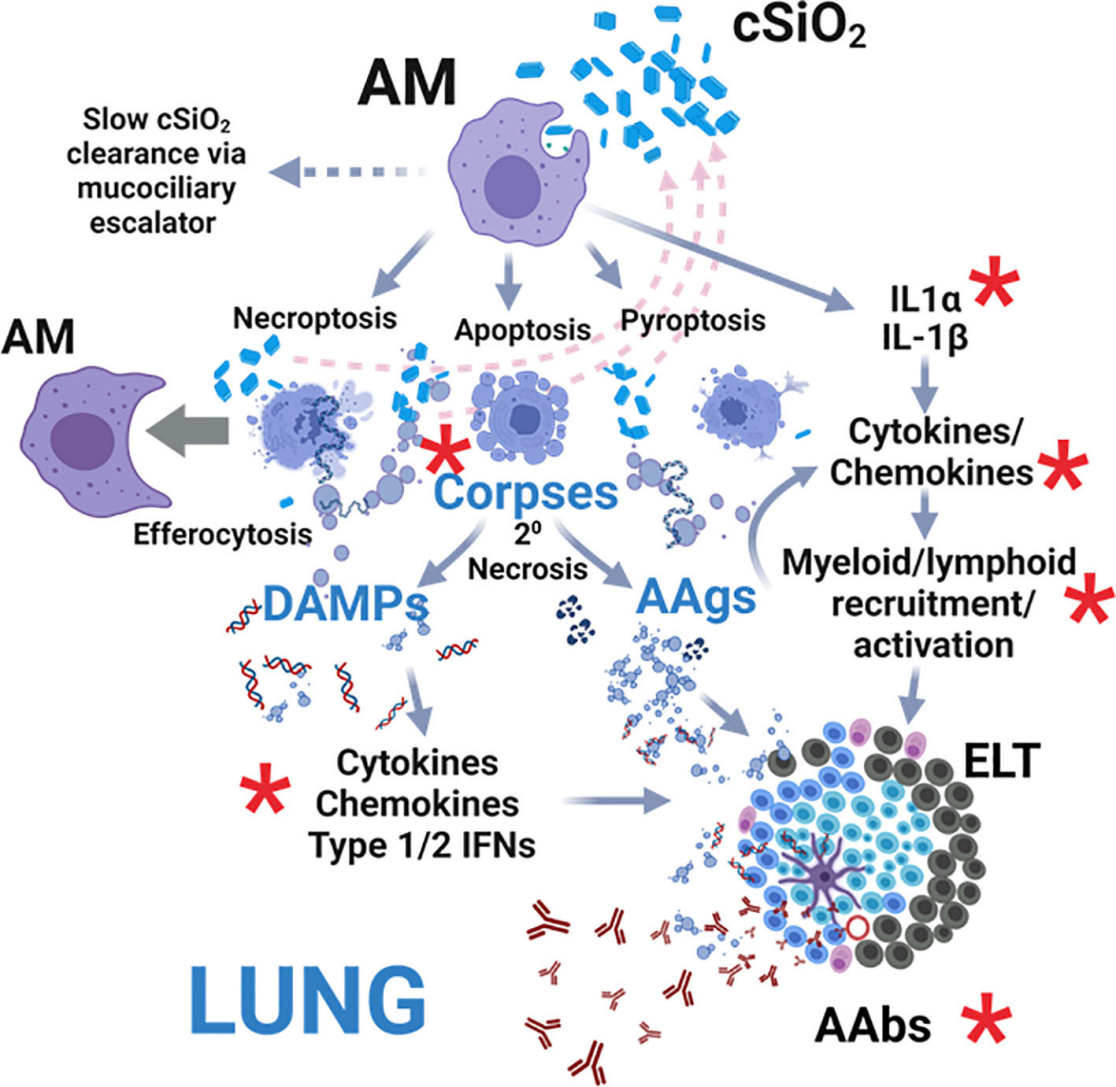
Hypothetical mechanisms for acute cSiO_2_-induced inflammation and autoimmunity targeted by DHA intervention. As first responders to inhaled particles, alveolar macrophages (AM) phagocytose cSiO_2_ and respond by 1) releasing IL-1α and IL-1β that drive further transcription, translation, and secretion of cytokines/chemokines promoting myeloid/lymphoid cell recruitment, 2) dying by necroptosis, apoptosis, and pyroptosis and releasing cSiO_2_ for further uptake (pink dashed arrows), and/or 3) sequestering cSiO_2_ and slowly clearing it from the lung via the mucociliary escalator. If resultant cell corpses are not immediately cleared by efferocytosis other AMs, they undergo secondary necrosis, releasing 1) damage-associated molecular patterns (DAMPs) that activate receptor-driven production of cytokines, chemokines, and IFNs and 2) autoantigens (AAgs) that promote antigen presentation and B/T cell activation. Collectively, these actions drive the development of pulmonary ectopic lymphoid tissues (ELT) that serve as sites for autoantibody (AAb) production against diverse AAgs, including nuclear, ribosomal, mitochondrial, and complement proteins. Red asterisks indicate targets of DHA suppression of early acute cSiO_2_-induced pulmonary inflammation, autoimmune-related gene expression, and AAb production in lupus-prone NZBWF1 mice that were identified in this investigation. Created with Biorender.com.

Acting as initial responders to inhaled foreign particles, AM phagocytose inhaled cSiO_2_, leading to: 1) release of IL-1α and IL-1β, driving cytokine/chemokine production and myeloid/lymphoid cell recruitment; 2) necroptosis, apoptosis, and pyroptosis, releasing cSiO_2_ for further uptake; and 3) sequestration and gradual clearance via the mucociliary escalator (29, 30) (**Figure 12**). In lupus-prone mice, uncleared cell corpses undergo secondary necrosis, releasing DAMPs and autoantigens (AAgs). DAMPs activate receptors, inducing cytokine, chemokine, and IFN production, while AAgs promote antigen presentation and B/T cell activation. Collectively, these events contribute to pulmonary ectopic lymphoid tissue (ELT) formation, loss of immunological tolerance, and autoantibody (AAb) production. We investigated here for the first time in lupus-prone NZBWF1 mice how dietary DHA impacts early (i.e., within 1 to 4 wk) transcriptional and autoantibody responses elicited in the lung by single acute cSiO_2_ exposure. DHA feeding modestly suppressed cSiO_2_-triggered elevations in total cell and lymphocyte counts in BALF as well cytokine/chemokine protein expression in the lung. Additionally, cSiO_2_ promoted more marked cell corpse accumulation and expression of inflammatory/autoimmunity-associated genes in the lungs of Con-fed than DHA-fed mice. Finally, by 28 d PI, cSiO_2_ induced more robust AAb responses for representative nuclear, ribosomal, mitochondrial, complement, and death-induced proteins in Con-fed mice than did DHA-fed mice. Since these findings were highly consistent with earlier observations in subchronic studies of DHA amelioration of cSiO_2_-induced autoimmunity (8–12), we further employed IPA and identified key upstream regulators comprising cytokines (TNF, IL-1β, IL-1α, IFNγ, IFNα), transmembrane receptors (IFNAR, TLR-3, TLR4, TLR7, TLR9), TLR adapters (MYD88, TICAM1), transcription factors (BHLHE40, CITED2, IRF7, NFκB, SPL1, and RELA) and a GTPase (Irgm1). As depicted in **Figure 12**, our findings support the premise that DHA suppression of both early cSiO_2_-induced dead cell accumulation and innate/adaptive immune gene expression in the lungs of NZBWF1 mice is associated with resilience to loss of immunological tolerance resulting in production of pathogenic AAbs.

Increasing the ω-3 PUFA content within cell membranes is a prerequisite for many of their anti-inflammatory and proresolving effects and was critical to the suppression of cSiO_2_-induced autoimmunity in the subchronic model (12). Specifically, we employed a DHA dose that was calorically equivalent to safe human consumption of 5 g/d that raised the ω-3 PUFA content in lungs from 4% of total fatty acids in Con-fed mice to 14% in the DHA-fed mice. These changes approximate those seen in the longer-term subchronic studies in the NZBWF1 model (6, 23). Furthermore, this percentage has been reported in persons consuming diets high in ω-3 PUFAs (12). ω-3 PUFAs can inhibit the metabolism of ω-6 PUFAs into proinflammatory eicosanoids, such as thromboxanes, prostaglandins, and leukotrienes (31). The lipid byproducts stemming from the arachidonic acid cascade primarily exhibit inflammatory actions, particularly during episodes of acute inflammation. Shifting the PUFA balance in favor of ω-3 PUFAs, in contrast to ω-6 PUFAs like arachidonic acid, has the potential to enhance the pro-resolving characteristics promoted by lipid mediators derived from ω-3s. A recent study has provided evidence of a strong correlation between the levels of ω-3 PUFAs in both plasma and red blood cells and the production of downstream lipid mediators (32). Similarly, supplementation with EPA and DHA resulted in a reduction in ω-6 PUFAs, particularly arachidonic acid, along with a decrease in ω-6 PUFA-derived metabolites. Consistent with the latter mechanism, we have recently demonstrated that DHA supplementation suppresses the induction of myriad cSiO_2_-induced eicosanoids in alveolar macrophages (33).

Besides competing with ω-6 PUFAs, ω-3 PUFAs can directly influence inflammatory pathways (34). For example, DHA and EPA can disrupt the activation of transmembrane receptors linked to inflammatory signaling by enhancing membrane fluidity and inhibiting the formation of lipid rafts (35). Also, both extracellular and intracellular phospholipases can cleave PUFAs from the cell membrane, and the resultant liberated DHA and EPA may stimulate transmembrane receptors or intracellular receptors known for their role in suppressing proinflammatory signaling (36, 37). Specifically, ω-3 PUFAs can counteract TLR activation (38, 39) and interfere with NF-kB-dependent transcription by activating PPARγ (40, 41). In line with this mechanism, we have shown that DHA suppresses cSiO_2_-induced inflammasome activation and IL-1 cytokine release in macrophages by acting at the level of TLR-mediated priming. Furthermore, both DHA and EPA undergo metabolism to generate specialized pro-resolving mediators (SPMs) like maresins, resolvins, protectins, and anti-inflammatory epoxide metabolites (32, 42). These SPMs effectively impede inflammatory signaling (43, 44) and facilitate the process of efferocytosis, crucial for the removal of deceased cells, a critical step in thwarting the pathogenesis of autoimmune diseases (45, 46).

Morphometry of DNA fragmentation by TUNEL provides a general measurement of dead cells that can include apoptosis, pyroptosis, and necrosis (47), all of which can be induced by cSiO_2_ in macrophages (48–50) (**Figure 12**). It is critical to note that cSiO_2_-induced TUNEL^+^ lung tissue was much more robust at 7 d than at 28 d PI (**Figure 4D**). It is possible that at the later time point, injurious particles may have been sufficiently sequestered/cleared to prevent further macrophage death and residual cell corpses removed by efferocytosis with net effect of reducing the TUNEL response. Nevertheless, the finding that cSiO_2_-induced TUNEL positivity was lower at 7 d PI in DHA-fed mice than those fed Con-diet suggests that ω-3 PUFA supplementation suppressed early dead cell accumulation. One explanation for this observation is that DHA suppresses cSiO_2_-induced macrophage death, which is consistent with our observation of reduced macrophage numbers at 7 d PI in bronchoalveolar lavage fluid from cSiO_2_-treated Con-fed mice but not DHA-fed mice. In further support of this idea, we previously reported that DHA suppressed cSiO_2_-induced death in different macrophage models, including primary alveolar macrophages (AM) isolated from NZBWF1 mice and RAW 264.7 murine macrophages (48). An alternative explanation for reduced TUNEL^+^ cells in the lungs of DHA-fed mice at 7 d PI is enhanced clearance by efferocytosis. Consistent with this notion, in a prior investigation, we found that effector macrophages could engulf target macrophage corpses elicited by apoptosis, pyroptosis, and necrosis but that preincubation of target macrophages with DHA prior to death induction significantly enhanced their efferocytosis by effector macrophages (48). Thus, DHA might be both capable of attenuating macrophage death and potentiating efferocytosis, with the net effect of reducing the accumulation of degrading cell corpses that ultimately activate inflammation and elicit autoimmunity.

Increased dead cells in the lung following cSiO_2_ instillation can further serve as a rich source of damage-associated molecular patterns (DAMPs), such as DNA and RNA that are capable of activating toll-like receptors (TLRs) and driving gene transcription (51). This premise gains reinforcement from the IPA findings represented in **Figure 9**, indicating that TLRs 3, 4, 7, and 9; TLR adapters MyD88 and TICAM1; transcription regulators NF-κB and RelA; as well as the cytokines TNF-α, IL-1α, and IL-1β stood out as the primary upstream regulators governing the cSiO_2_-induced gene expression in the lungs of mice fed with the control diet at both 7 d and 28 d. Moreover, IPA identified other upstream activators likely critical in the cSiO_2_-triggered Type 1 IFN (IFNAR, IRF-7, and IFNα) and Type 2 IFN (IFNγ) responses observed here and known to be associated with human lupus (52, 53). Significantly, the activation Z scores for all these upstream regulators were substantially lower in the lungs of DHA-fed mice, suggesting that they could be critical targets of ω-3 PUFAs.

In addition to activation, we identified two top upstream regulators with high Z scores predictive of inhibitory actions in cSiO_2_-treated Con-fed mice. These Z scores were markedly improved in cSiO_2_-treated DHA-fed mice. One of these is immunity-related GTPase family M protein 1 (Irgm1), an IFN-inducible cytoplasmic GTPase that regulates autophagy and mitochondrial homeostasis (54). Irgm1 is known to dually modulate conserved self- and other-directed immune responses at the host-environment interface. Interestingly, Irgm1–/– mice exhibit lymphocytic infiltration in lung and other mucosal tissues with the production of AAbs, including anti-Ro and anti-La, which were seen in our protein array results. The second inhibitory regulator is a CBP/p300-interacting transactivator with glutamic acid/aspartic acid-rich carboxyl-terminal domain 2 (CITED2) (55). CITED2 is a critical intrinsic negative regulator of inflammation that broadly reduces proinflammatory gene programs in macrophages.

The presence of immunogenic dying or dead cells in the lung following acute cSiO_2_ instillation in the context of a proinflammatory cytokine-, chemokine-, and IFN-rich milieux could drive AAg presentation and AAb production in the lung (48, 56). Consistent with this notion, cSiO_2_ elicited both ELT neogenesis as well as a wide range of AAbs with specificities for nuclear, ribosomal, mitochondrial, and complement proteins. We found that these AAb responses were markedly suppressed in the DHA cohort. Furthermore, we qualitatively confirmed a potential role of dead cells by showing cSiO_2_ induction and DHA suppression of IgG Abs in BALF reactive with supernatants of cSiO_2_-and staurosporine-killed Mph as well as purified nucleosomes. It is noteworthy that the activation of TLR 7 and IFNAR due to cSiO_2_ could be closely connected to the AAb production. It is established that type 1 IFNs and TLR7 stimulants play a role in triggering the activation and class change of autoreactive transitional B cells (57). Intriguingly, autoreactive B cells in the transitional phase are typically regulated by tolerance mechanisms. The concurrent rise in the presence of nucleic acid-containing antigens and type 1 IFNs could potentially disrupt this regulatory checkpoint in lupus.

One limitation of this work and our prior studies is that we did not identify specific cell phenotypes that were responsible for the upregulated autoimmune gene responses. For example, while alveolar macrophages are primary first responders to inhaled cSiO_2_ and, therefore, likely responsible for many of the responses observed here at 7 d PI. However, it is possible that elevated chemokines drive the recruitment of additional monocytes/macrophages to the alveolar space by 28 d PI. It might be further speculated that DHA feeding skews these macrophages from the M1 proinflammatory phenotype to the M2 anti-inflammatory phenotype. Furthermore, other cell types that contribute to cSiO_2_-indued pulmonary transcriptional and autoantibody responses could be recruited such as neutrophils, dendritic cells, B/T lymphocytes, cells, and Type 1 and 2 epithelial cells. Future investigations employing single-cell RNAseq could be very informative in delineating the affected phenotypes contributing to cSiO_2_-triggered inflammation and DHA’s ameliorative effects. A further limitation of this study was that restricting our phenotype characterization of ELT cells using pan T cell (CD3^+^) and B cell (CD45R^+^) antibodies made it challenging to discriminate subtle effects of cSiO_2_ and DHA on different phenotypes within these two broad populations. Additional studies are therefore needed using multicolor immunofluorescence in conjunction with confocal microscopy/flow cytometry to resolve quantitative changes in differentiated T and B lymphocytes and the appearance of other cell types that contribute to germinal center development AAb production, such as follicular dendritic cells.

## 5 Conclusion

The results presented here provide novel mechanistic insights into how translationally relevant DHA supplementation suppresses early (i.e., within 1 to 4 wk PI) aberrant acute cSiO_2_-induced lung lymphocyte recruitment, immunogenic cell corpse accumulation, IFN-regulated gene responses, and AAb production in lupus-prone mice. Advantageous features of the acute model used here are that it is logistically much simpler and faster than the subchronic model employed previously (1 month vs. 4 months, respectively). Consequently, this surrogate model holds the potential for further mechanistic dissection into how alterations in the cellular lipidome impact loss of immunological tolerance triggered by cSiO_2_ and other environmental agents in genetically predisposed mice.

## Data availability statement

The datasets presented in this study can be found in online repositories. The names of the repository/repositories and accession number(s) can be found below: https://datadryad.org/stash, https://doi.org/10.5061/dryad.5mkkwh79g.

## Ethics statement

The animal study was approved by Michigan State University Institutional Animal Care and Committee (AUF # PROTO201800113). The study was conducted in accordance with the local legislation and institutional requirements.

## Author contributions

PC: Data curation, Formal analysis, Investigation, Methodology, Project administration, Validation, Visualization, Writing – original draft, Writing – review & editing. AB: Conceptualization, Data curation, Formal analysis, Methodology, Visualization, Writing – original draft, Writing – review & editing. OF: Data curation, Formal analysis, Methodology, Visualization, Writing – original draft, Writing – review & editing. JW: Conceptualization, Formal analysis, Investigation, Methodology, Writing – review & editing. RL: Formal analysis, Investigation, Methodology, Supervision, Visualization, Writing – original draft, Writing – review & editing. LR: Formal analysis, Investigation, Methodology, Visualization, Writing – review & editing. QL: Formal analysis, Investigation, Methodology, Resources, Writing – review & editing. JH: Conceptualization, Formal analysis, Investigation, Methodology, Resources, Visualization, Writing – original draft, Writing – review & editing. JP: Conceptualization, Funding acquisition, Methodology, Project administration, Resources, Supervision, Writing – original draft, Writing – review & editing.
