# Supplementary material for: Dietary docosahexaenoic acid supplementation inhibits acute pulmonary transcriptional and autoantibody responses to a single crystalline silica exposure in lupus-prone mice
Source: Front Immunol. 2024 Feb 1;15:1275265. doi: 10.3389/fimmu.2024.1275265 (PMC10867581; doi:10.3389/fimmu.2024.1275265)
Supplement: Supplementary file 1 [file DataSheet_1.zip › Supplementary data table and figures.pdf]

Supplementary Material

Dietary Docosahexaenoic Acid Supplementation Inhibits Acute Pulmonary Transcriptional and Autoantibody Responses to a Single Crystalline Silica Exposure in Lupus-Prone Mice

Preeti S Chauhan, Abby D Benninghoff , Olivia K Favor, James G Wagner, Ryan P Lewandowski, Lichchavi D. Rajasinghe, Quan-Zhen Li, and Jack R Harkema, James J Pestka[†]

[†] Correspondence: James Pestka, pestka@msu.edu

Supplemental Table 1. Fatty acid content of representative experimental diets

Supplementary Figure 1. DHA supplementation does not influence $cSiO_2$-induced lung inflammation

Supplementary Figure 2. DHA supplementation does not influence $cSiO_2$-induced CD3+ and CD45R+ cell accumulation in the lung.

Supplementary Figure 3. Ingenuity pathway depicting TNF as an upstream regulator of $cSiO_2$-induced gene network at 7 d PI

Supplementary Figure 4. Ingenuity pathway depicting IL-1β as an upstream regulator of $cSiO_2$-induced gene network at 7 d PI.

Supplementary Figure 5. Ingenuity pathway depicting IFNAR as an upstream regulator of $cSiO_2$-induced gene network at 7 d PI.

Supplementary Figure 6. Ingenuity pathway depicting IFNγ as an upstream regulator of $cSiO_2$-induced gene network at 7 d PI.

Supplementary Figure 7. DHA supplementation suppresses cSiO2-triggered upstream regulator associated gene expression

Supplementary Figure 8. Heat map depicting Ab-score for all AAbs of IgG isotype in BALF at 28 d PI

Supplemental Table 1. Fatty acid content of representative experimental diets[a]

Common Name	Formula	Con	DHA
Myristic	C14:0	0.31 ± 0.00	3.38 ± 0.56
Palmitic	C16:0	4.46 ± 0.00	5.86 ± 0.30
Palmitoleic	C16:1ω7	0.07 ± 0.00	0.70 ± 0.11
Stearic	C18:0	1.59 ± 0.00	1.27 ± 0.03
Elaidic	C18:1t	0.17± 0.00	0.11 ± 0.01
Oleic	C18:1ω9	49.05 ± 0.06	36.81 ± 2.42
Linoelaidic	C18:2ω6t	0.09 ± 0.00	0.08 ± 0.00
Linoleic	C18:2ω6	12.96 ± 0.06	10.08 ± 0.36
Arachidic	C20:0	0.27 ± 0.00	0.20 ± 0.01
gamma-Linolenic	C18:3ω6	0.01 ± 0.00	0.01 ± 0.00
Eicosenoic	C20:1ω9	019 ± 0.00	0.15 ± 0.01
alpha-Linolenic	C18:3ω3	0.23 ± 0.01	0.19 ± 0.00
Eicosadienoic	C20:2ω6	0.03 ± 0.00	0.04 ± 0.01
Behenic	C22:0	0.19 ± 0.00	0.17 ± 0.00
Arachidonic	C20:4ω6	0.00 ± 0.00	0.01 ± 0.00
Lignoceric	C24:0	0.13 ± 0.00	0.11 ± 0.00
Eicosapentaenoic	C20:5ω3	0.00 ± 0.00	0.01 ± 0.00
Nervonic	C24:1ω9	0.11 ± 0.00	0.07 ± 0.00
Docosapentaenoic ω3	C22:5ω3	0.00 ± 0.00	0.17 ± 0.03
Docosahexaenoic	C22:6ω3	0.00 ± 0.00	10.59 ± 1.82
∑ SFA		9.92 ± 0.01	10.82 ± 0.82
∑ MUFA		70.77 ± 0.09	37.85 ± 2.32
∑ ω-6 PUFA		18.70 ± 0.08	10.21 ± 0.35
∑ ω-3 PUFA		0.34 ± 0.00	10.96 ± 1.85
ω 6: ω 3 ratio		55.38 ± 0.38	0.95 ± 0.19

[a]Data presented as percent of total fatty acids as measured by GLC. [b]Data are mean ± SEM, n=2.

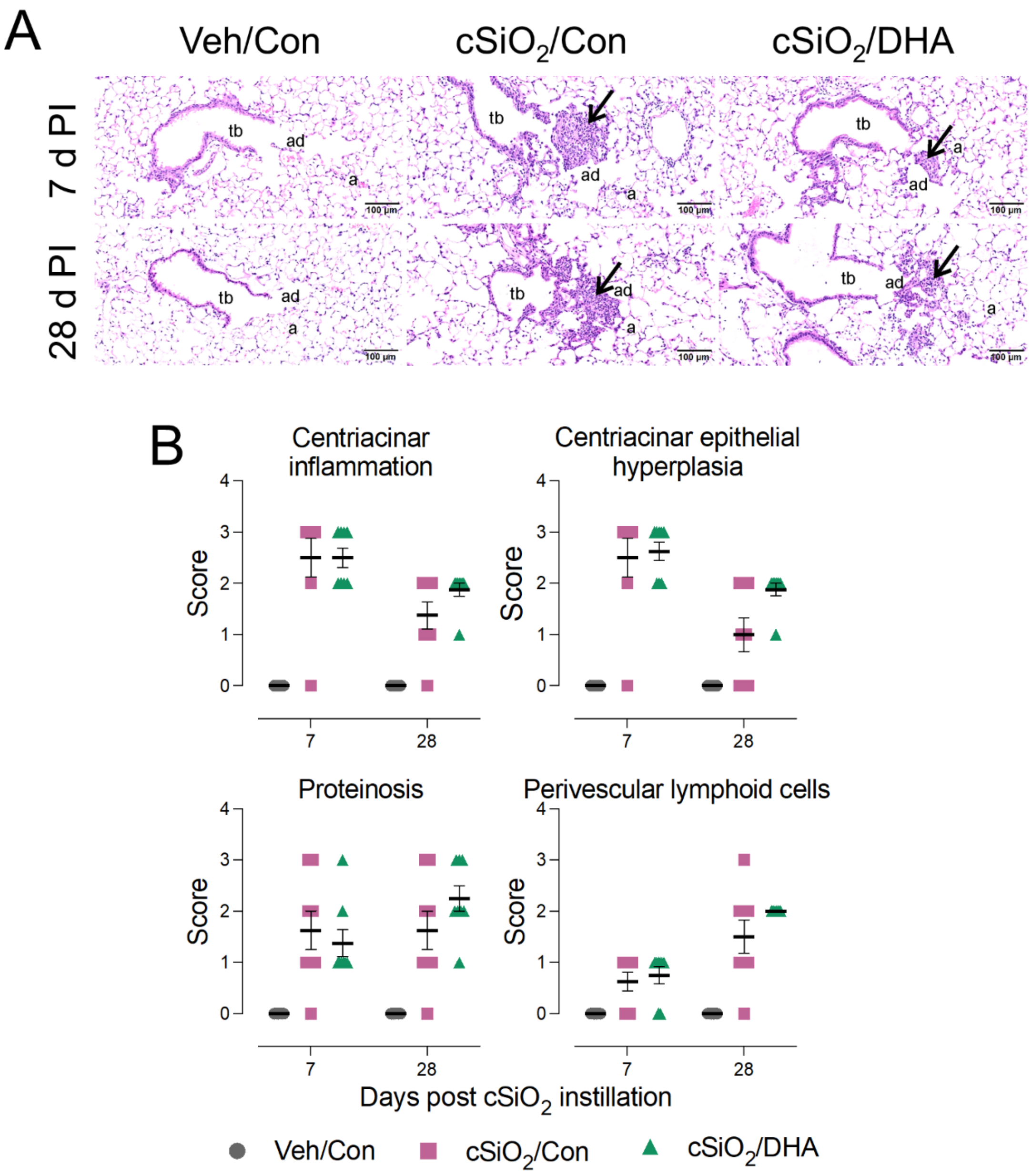

Supplementary Figure 1. DHA supplementation does not influence $cSiO_2$-induced lung inflammation. A) Light photomicrographs of hematoxylin and eosin-stained lung tissue sections illustrating centriacinar regions from vehicle instilled/control diet fed mice, $cSiO_2$-instilled/control diet fed mice and $cSiO_2$-instilled/DHA-supplemented diet fed mice, 7d and 21d post-instillation. Centriacinar inflammation, alveolar macrophage/monocyte accumulation and alveolar type 2 epithelial hyperplasia is present predominately in proximal alveolar ducts (arrows) of lung sections of $cSiO_2$/Con and $cSiO_2$/DHA mice, with greatest lesion severity in $cSiO_2$/Con mice at 7d post-instillation. Persistent but attenuated centriacinar lesions are present after with DHA supplemented diet and increase time after instillation (28d). B) Graphical presentations of semi-quantitative severity scores for specific centriacinar lesions (inflammation, epithelial hyperplasia) as well as alveolar proteinosis and perivascular lymphoid cell accumulation. See text for further details of criteria used for numerical scoring. tb, terminal bronchiole; ad, alveolar duct; a, alveolar parenchyma

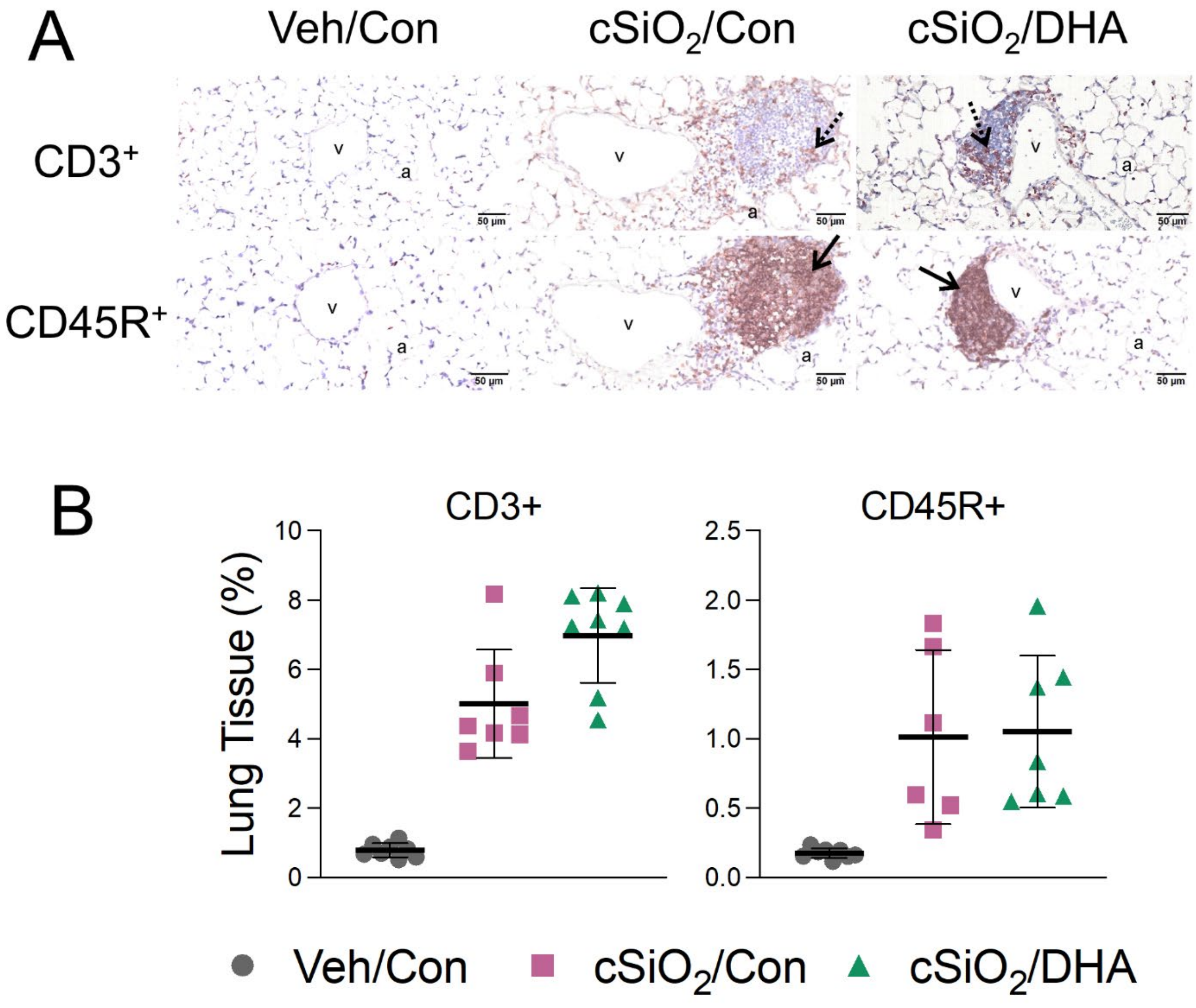

Supplementary Figure 2. DHA supplementation does not influence $cSiO_2$-induced CD3+ and CD45R+ cell accumulation in the lung. A) Light photomicrographs of lung sections immunohistochemically stained for perivascular CD3+ and CD45R+ lymphoid cells, T and B cells respectively (brown chromagen; arrows). Tissue sections, countered stained with hematoxylin, were taken from lungs of vehicle instilled/control diet fed mice, $cSiO_2$-instilled/control diet fed mice and $cSiO_2$-instilled/DHA-supplemented diet fed mice, 21d post-instillation. B) Graphical presentations of semiquantitative severity scores of perivascular accumulation of CD3+ and CD45R+ lymphoid cells that increased with $cSiO_2$ instillations but were statistically unaffected by DHA dietary supplementation.

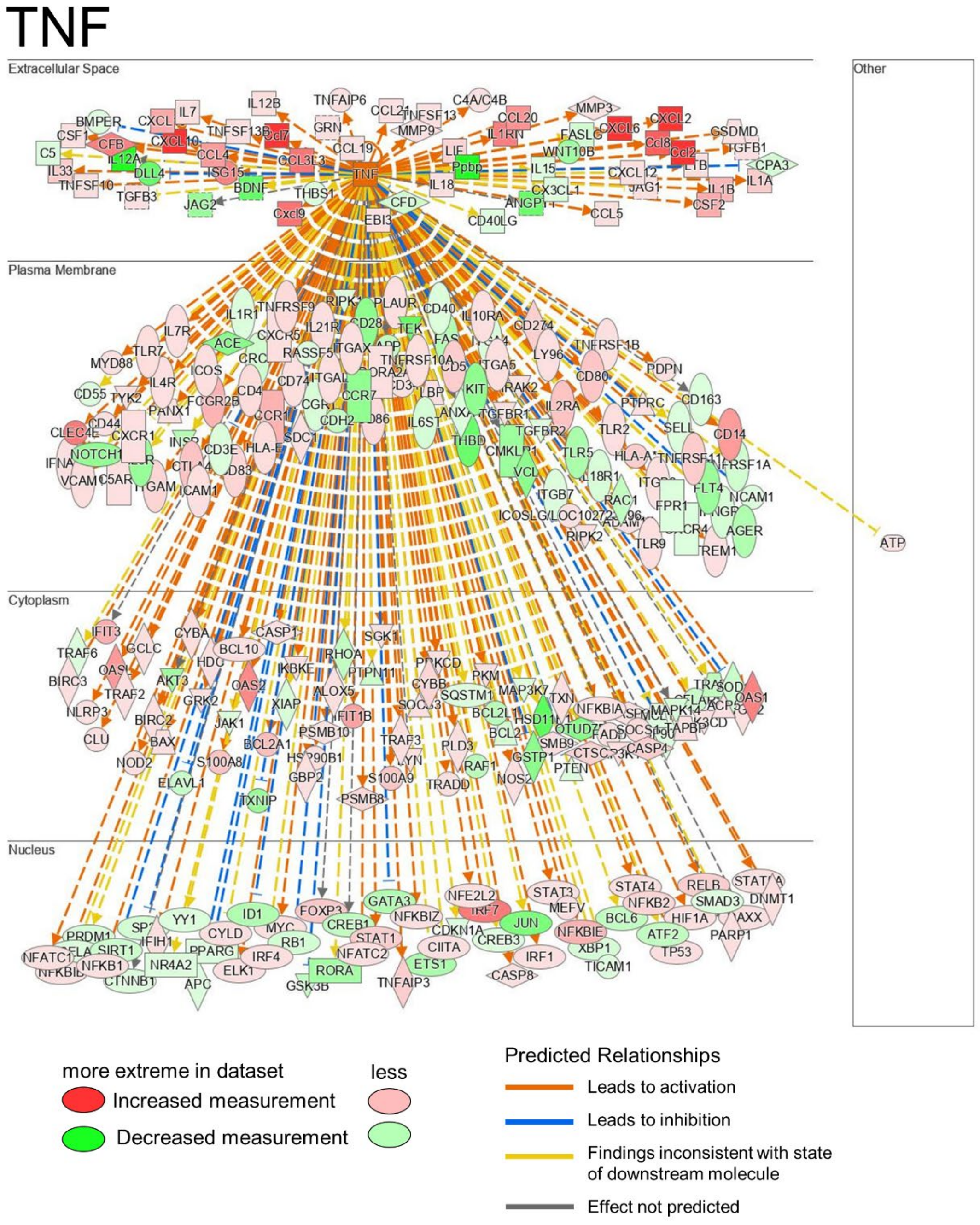

Supplementary Figure 3. Ingenuity pathway depicting TNF as an upstream regulator of $cSiO_2$-induced gene network at 7 d PI. Ingenuity Pathway Analysis (IPA) gene network for TNF in cSiO2-instllled mice fed control diet at 7 d PI. Downstream gene heat map reflect increased (shades of red) or decreased (shades of green) expression. Lines show predicted inhibition (blue) or activation (orange) of the downstream genes. Yellow and gray lines depict inconsistent finding according to IPA

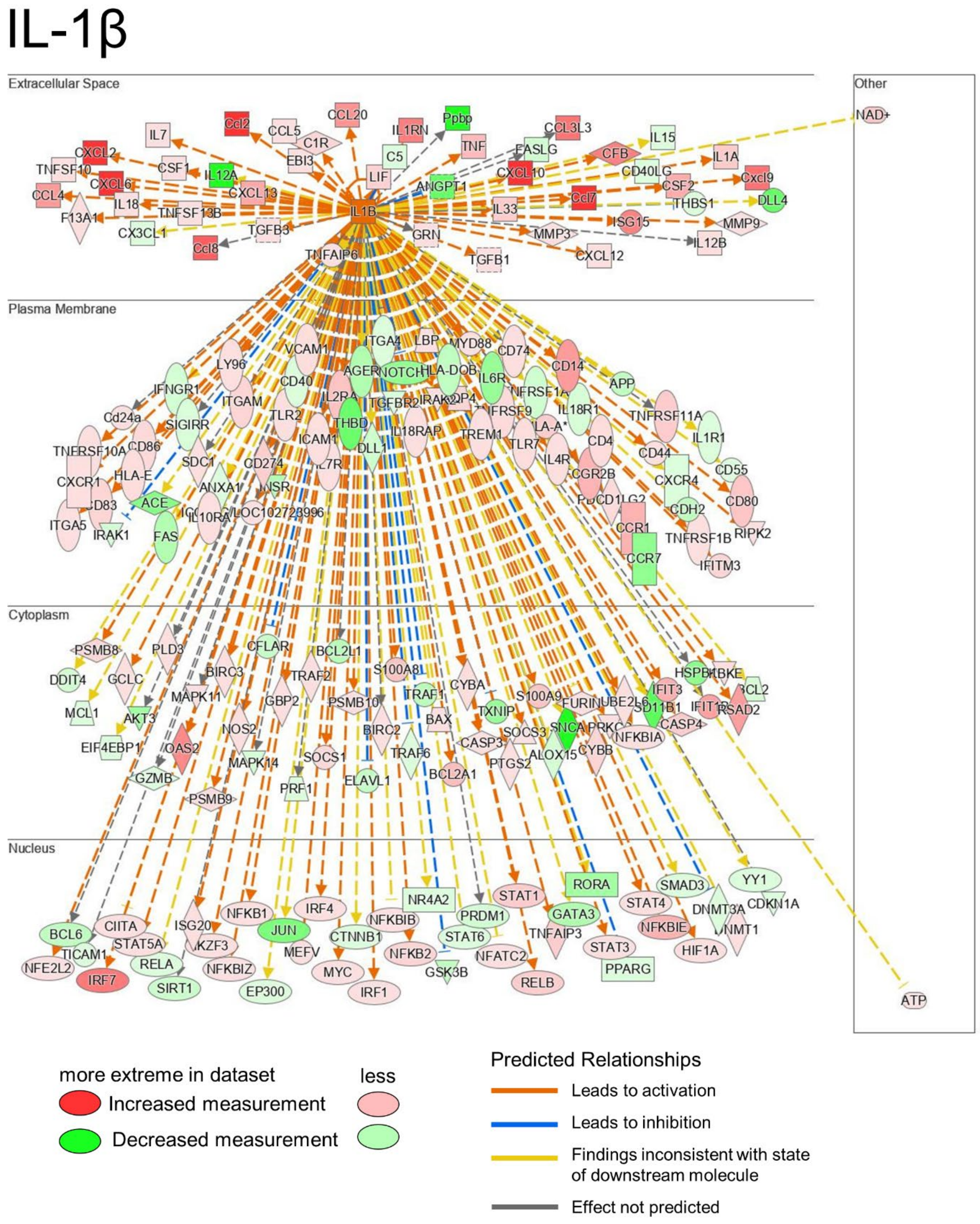

Supplementary Figure 4. Ingenuity pathway depicting IL-1β as an upstream regulator of $cSiO_2$-induced gene network at 7 d PI. Ingenuity Pathway Analysis (IPA) gene network for IL-1β in $cSiO_2$-instllled mice fed control diet at 7 d PI. Downstream gene heat map reflect increased (shades of red) or decreased (shades of green) expression. Lines show predicted inhibition (blue) or activation (orange) of the downstream genes. Yellow and gray lines depict inconsistent finding according to IPA.

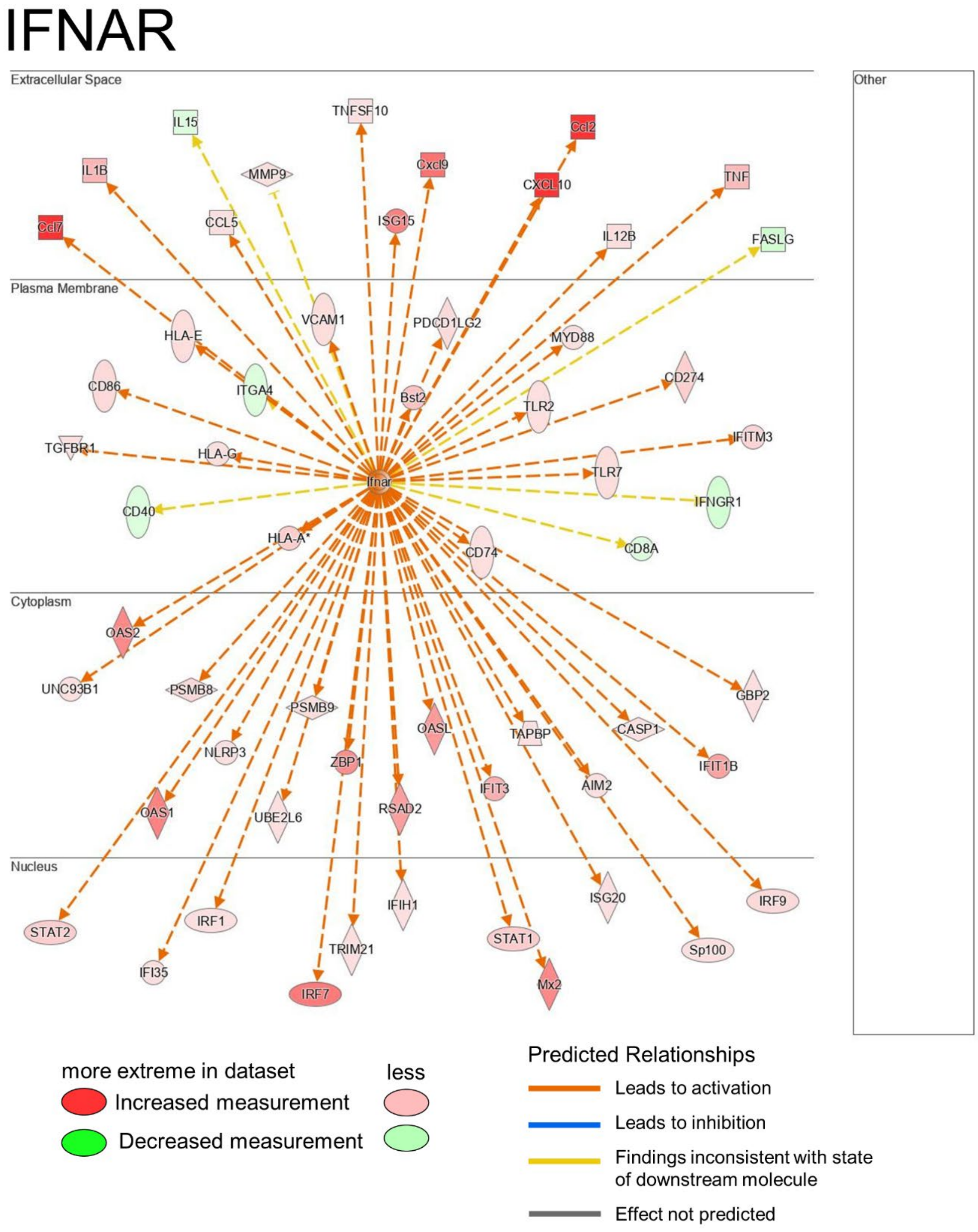

Supplementary Figure 5. Ingenuity pathway depicting IFNAR as an upstream regulator of $cSiO_2$-induced gene network at 7 d PI. Ingenuity Pathway Analysis (IPA) gene network for IFNAR in $cSiO_2$-instllled mice fed control diet at 7 d PI. Downstream gene heat map reflect increased (shades of red) or decreased (shades of green) expression. Lines show predicted inhibition (blue) or activation (orange) of the downstream genes. Yellow and gray lines depict inconsistent finding according to IPA.

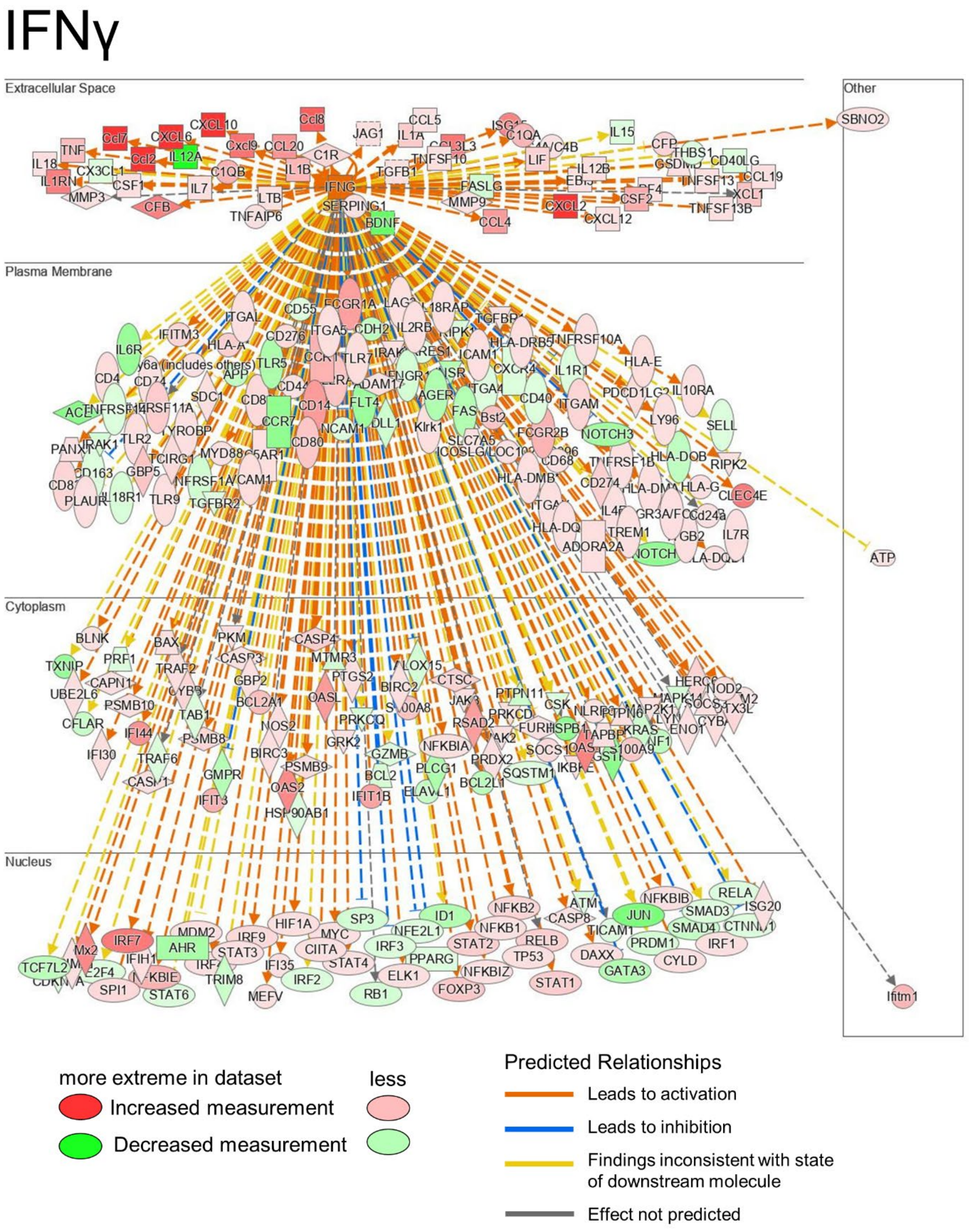

Supplementary Figure 6. Ingenuity pathway depicting IFNγ as an upstream regulator of cSiO$_2$-induced gene network at 7 d PI. Ingenuity Pathway Analysis (IPA) gene network for IFNγ in cSiO$_2$-instllled mice fed control diet at 7 d PI. Downstream gene heat map reflect increased (shades of red) or decreased (shades of green) expression. Lines show predicted inhibition (blue) or activation (orange) of the downstream genes. Yellow and gray lines depict inconsistent finding according to IPA.

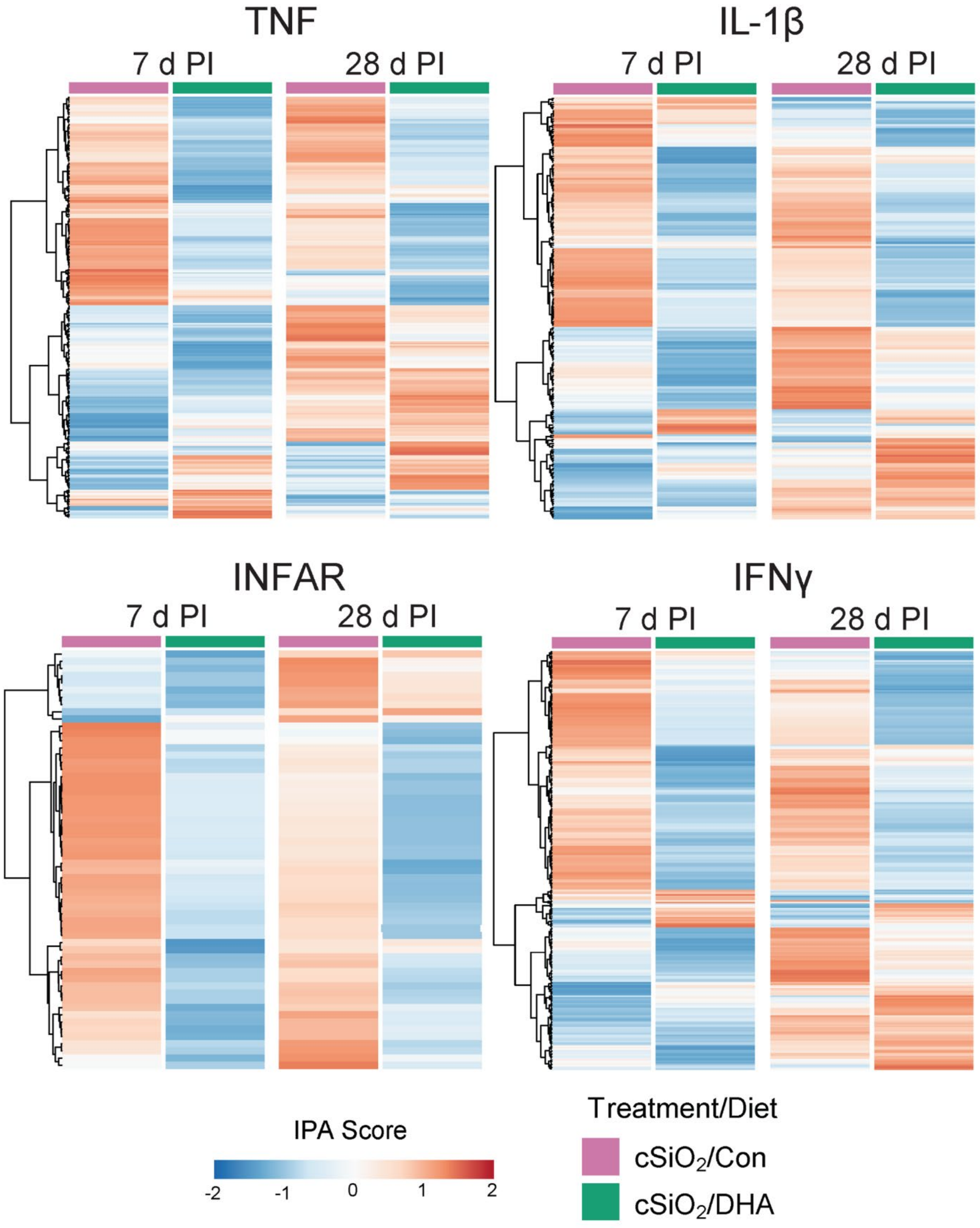

Supplementary Figure 7. DHA supplementation suppresses cSiO2-triggered upstream regulator associated gene expression. (A) TNF, (B) Ifnar (C) IFNAG and (D)IL-1b associated genes heatmap for $cSiO_2$/Con and $cSiO_2$/DHA identified by Ingenuity Pathway Analysis (IPA) upstream regulators analysis. Values are shown as the row centered, variance stabilized IPA score. Rows are clustered using Euclidean distance and Ward linkage.

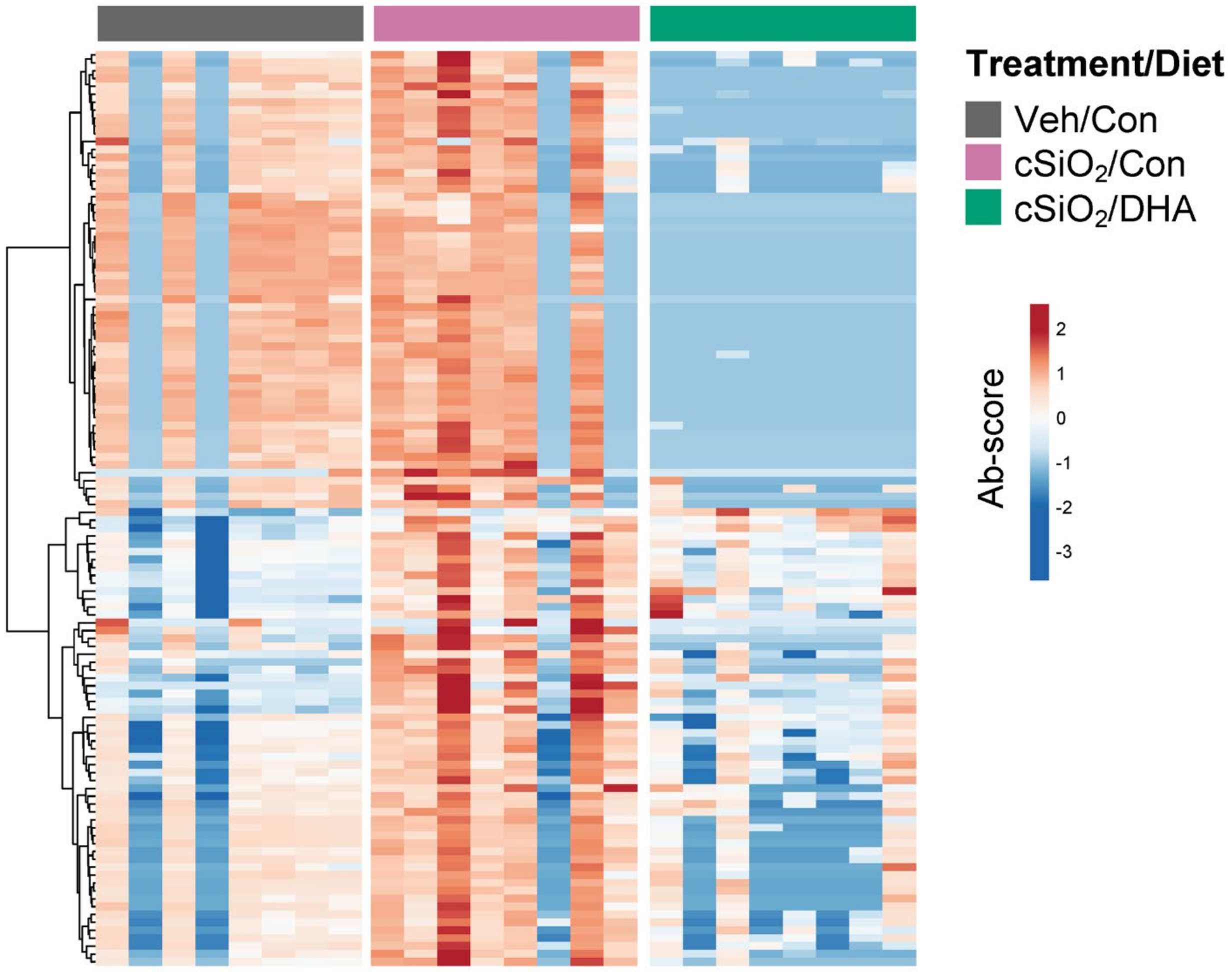

Supplementary Figure 8. Heat map depicting Ab-score for all AAbs of IgG isotype in BALF at 28 d PI. Heat maps depict Ab-score values (row centered, variance stabilized) for expression of all AAbs of the IgG isotype in BALF at 28 d PI as determined by microarray. Rows are clustered using Euclidean distance and Ward linkage.
